# Cancer Nanopharmaceuticals: Physicochemical Characterization and In Vitro/In Vivo Applications

**DOI:** 10.3390/cancers13081896

**Published:** 2021-04-15

**Authors:** Aleksandra Zielińska, Marlena Szalata, Adam Gorczyński, Jacek Karczewski, Piotr Eder, Patrícia Severino, José M. Cabeda, Eliana B. Souto, Ryszard Słomski

**Affiliations:** 1 Institute of Human Genetics, Polish Academy of Sciences, Strzeszyńska 32, 60-479 Poznań, Poland; szalata@up.poznan.pl (M.S.); ryszard.slomski@up.poznan.pl (R.S.); 2 Department of Pharmaceutical Echnology, Faculty of Pharmacy, University of Coimbra, Pólo das Ciências da Saúde, Azinhaga de Santa Comba, 3000-548 Coimbra, Portugal; 3 Department of Biochemistry and Biotechnology, Poznań University of Life Sciences, Dojazd 11, 60-632 Poznań, Poland; 4 Faculty of Chemistry, Adam Mickiewicz University, Uniwersytetu Poznańskiego 8, 61-614 Poznań, Poland; adam.gorczynski@amu.edu.pl; 5 Department of Environmental Medicine, Poznan University of Medical Sciences, 61-701 Poznan, Poland; jacek_karczewski@yahoo.com; 6 Department of Gastroenterology, Dietetics and Internal Diseases, Poznan University of Medical Sciences, Przybyszewskiego 49, 60-355 Poznań, Poland; piotreder@ump.edu.pl; 7 Center for Biomedical Engineering, Department of Medicine, Brigham and Women & Hospital, Harvard Medical School, 65 Landsdowne Street, Cambridge, MA 02139, USA; patricia_severino@itp.org.br; 8 Biotechnological Postgraduate Program, Institute of Technology and Research (ITP), Nanomedicine and Nanotechnology Laboratory (LNMed), University of Tiradentes (Unit), Av. Murilo Dantas 300, Aracaju 49010-390, Brazil; 9 Tiradentes Institute, 150 Mt Vernon St, Dorchester, MA 02125, USA; 10 ESS-FP, Escola Superior de Saúde Fernando Pessoa, Rua Delfim Maia 334, 4200-253 Porto, Portugal; jcabeda@ufp.edu.pt; 11 FP-ENAS-Fernando Pessoa Energy, Environment and Health Research Unit, Universidade Fernando Pessoa, Praça 9 de Abril, 349, 4249-004 Porto, Portugal; 12 CEB–Centre of Biological Engineering, Campus de Gualtar, University of Minho, 4710-057 Braga, Portugal

**Keywords:** nanoparticles, cancer diagnosis, treatment of cancer, nanotherapeutics, therapeutic nanoparticles

## Abstract

**Simple Summary:**

The use of nanopharmaceuticals in chemotherapeutic regimens has become a promising approach for the treatment of most of the demanding types of cancers. Their in-depth physicochemical characterization plays an instrumental role in the quality control of a promising new medicine. The combined function of diagnosis and therapy in the same nanopharmaceuticals created the so-called nanotheragnostics, which have found a broad range of applications in this field. This review addresses the state of the art on the use of nanopharmaceuticals in cancer therapy and the latest challenges encountered in regulating these new medicines.

**Abstract:**

Physicochemical, pharmacokinetic, and biopharmaceutical characterization tools play a key role in the assessment of nanopharmaceuticals’ potential imaging analysis and for site-specific delivery of anti-cancers to neoplastic cells/tissues. If diagnostic tools and therapeutic approaches are combined in one single nanoparticle, a new platform called nanotheragnostics is generated. Several analytical technologies allow us to characterize nanopharmaceuticals and nanoparticles and their properties so that they can be properly used in cancer therapy. This paper describes the role of multifunctional nanoparticles in cancer diagnosis and treatment, describing how nanotheragnostics can be useful in modern chemotherapy, and finally, the challenges associated with the commercialization of nanoparticles for cancer therapy.

## 1. Introduction

The continuous breakthrough we are witnessing in the field of nanomedicine confirms the wide use of nanoparticles for a range of advanced therapies (e.g., radiation, cell therapy). A nanoparticle can be defined as any material that is used in the formulation of a drug resulting in a final product smaller than 1 micron in size. The use of nanoparticles for imaging analysis is especially promising in cancer diagnosis because they allow rapid recognition of tumor-related molecules [1,2]. Nanotheragnostics have already shown breakthroughs in the detection, diagnosis, and treatment of cancer. Together with thermal therapy, nanotheragnostics are one of the most significant approaches for cancer diagnosis, as they combine in the same device the capacity to diagnose and to deliver the chemotherapeutic drug at the site of action [3]. For this purpose, different types of nanoparticles can be used to simultaneously carry a drug and an imaging counterpart. Highly effective therapeutic agents can detect molecular changes at the early stage of tumor cells. Thanks to cancer nanotheragnostics that can be applied for simultaneous delivery of a few therapeutic molecules, it is possible not only to change the pharmacokinetics of drugs but also to enhance the therapeutic markers and reduce systemic toxicity thereby [4].

A nanopharmaceutical refers to a pharmaceutical agent formulated at the nanoscale (either within a nanoparticle or in form of drug nanocrystal), which can be further presented to the patient as tablets, capsules, ointments or creams, liquid suspensions, or even as aerosols. Nanoparticles are smart transport systems that can carry tumor-targeted drugs, which are either encapsulated inside a core or fixed onto the surface of the particles [5,6]. Commonly used nanoparticles, known as efficient carriers of cancer drugs may include: solid lipid nanoparticles and nanostructured lipid carriers [7], nanoemulsions [8], self-assembled nanostructures [9,10], polymeric nanoparticles [11,12,13], hybrid protein-inorganic nanoparticles [14], mesoporous silica nanoparticles [15], carbon nanotubes [16], and metal-derived nanoparticles [5,17]. Nanocrystals are described as solid crystallized drugs stabilized in an aqueous dispersion using a suitable surfactant [18,19].

Nanoparticles are used in cancer therapy because they contribute to widening the therapeutic window of the anticancer drug by increasing its bioavailability and reducing the risk of systemic toxicological effects attributed to site-specific targeting. In the biomedical scope, iron oxide nanoparticles are especially promising for targeting strategies [20,21]. Metallic and semiconductor nanoparticles have a major impact on cancer diagnosis and therapy and inhibit tumor growth. Scientific evidence exists on the great impact of magnetic iron oxide nanoparticles in preclinical and clinical research [22], in particular, given their biodegradable character associated with their potential use in disease imaging and tracing [21].

## 2. Physicochemical Characterization

**Figure 1:**
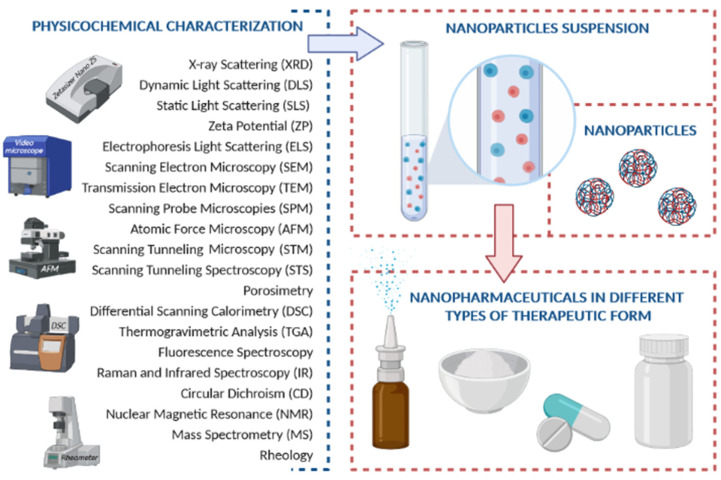
Graphical summary of the physicochemical methods utilized for the characterization of nanopharmaceuticals [own drawing].

Nanopharmaceuticals require an extensive physicochemical characterization for a range of properties, to be used in preclinical and clinical studies [23]. The same property should be evaluated by different techniques as the strengths and limitations of each technique compromise the choice of the most suitable method, while often a combinatorial characterization approach is needed [24]. Besides, their characterization needs to be reproducible so that the biomedical uses of nanotherapeutics can be reliable [25]. A selection of physicochemical methods commonly utilized for the characterization of nanopharmaceuticals is shown in Figure 1.

### 2.1. X-ray Scattering

XRD diffraction is used to analyze crystalline or polycrystalline materials and can be considered a primary tool for resolving tertiary structures of crystalline materials on an atomic scale [26]. In this technique, the sample is exposed to a collimated X-ray beam, with scattering intensity across parallel atomic planes stacked from the sample at specific angles. X-ray diffraction can also simply be described as the reflection of a collimated beam of X-rays incident on the crystalline planes of a sample examined according to Bragg’s law [27].

This technique is the main one used to assess the crystallinity of drugs, carriers, and excipients. It is also able to identify the crystalline phases of contaminants in drug synthesis processes. In contrast to XRD, whose applications are limited to crystalline materials, Small-Angle X-ray Scattering (SAXS) provides information of various characteristics (e.g., particles’ size, shape, surface-to-volume ratio, distribution) by examining crystalline or amorphous polymer materials, proteins to nanomaterials [28]. SAXS has been widely explored as a powerful technique for investigating material structures in length scales ranging from 1 to 100 nm [29]. In this method, a portion of an incident X-ray beam, elastically dispersed from the sample forms a scattering pattern on an X-ray detector in the two-dimensional plane, perpendicular to the direction of the incident X-ray beam. By analyzing the intensity of the scattered X-ray collected within the scattering angle, SAXS can assess the distribution, size, shape, orientation, and structure of a variety of polymers and systems of bio-conjugated nanomaterials in solution [30]. It can also be separated into two techniques, depending on the nature of the radiation employed: small-angle neutron scattering and small-angle light scattering [31]. The features of angle dispersion in SAXS lead to the ability to study structures that are not repeated. Thus, perfect crystallized structures are not necessary, which simplifies sample preparation and makes SAXS a non-destructive method.

### 2.2. Dynamic Light Scattering (DLS)

This technique is based on two factors: Brownian motion and light scattering [32]. Brownian motion is defined as the random movement of solid particles in a fluid, liquid or gas, as a result of interactions between all atoms present in the same fluid. It has a direct proportionality relationship with temperature and an inverse proportionality relationship with the particle diameter and viscosity [33]. In the DLS technique, the particles are illuminated by a laser causing the light to be scattered. Then, fluctuations in scattered light intensity are measured at various angles, which are much faster when the particles are small in size. The correlation function indicates how long a particle has been in the same position [34]. This also depends on the size of the particles, the smaller, the greater the speed of movement and, therefore, a faster decay of function [29]. Through the Stokes-Einstein equation it is possible to calculate the effective/hydrodynamic diameter of the particles in thermal equilibrium with the solvent (Equation (1)):

d(H) = kT/3ΠηD,(1)

where d means hydrodynamic diameter, D is the translational diffusion coefficient (obtained through the correlation function), K is Boltzmann’s constant, T means a temperature, and ɳ describes viscosity.

### 2.3. Static Light Scattering (SLS)

In static light scattering (SLS), the intensity of detected light is averaged over time, which provides information on the particles’ molecular weight as well as their gyration radius (*R_g_*) [35]. The technique can be further developed through a simultaneous collection of data from multiple angles and allows insight into the scattering as a function of time and is termed multi-angle light scattering (MALS) [36,37]. This rendered it useful in combination with gel permeation chromatography (GPC) allowing characterization of macromolecular materials after their elution from the size exclusion columns [38].

### 2.4. Zeta Potential (ZP) and Electrophoresis Light Scattering (ELS)

The surface electrical charge on the shear surface is called the Zeta Potential (ZP), which is usually determined by measuring the velocity of the charged species towards the electrode in the presence of an external electric field in the sample solution, being a fundamental characteristic of the particle that can be quickly measured using light scattering techniques [29]. In an ionic solution, the surface of a charged particle is firmly attached to charged ions oppositely, forming a thin liquid layer called the ‘Stern layer’. This layer is surrounded by another one, this time diffuse and more external, which is made up of weakly associated ions [39]. The combination of these two layers results in the so-called double electrical layer. The double electric layer contains mobile ions and counter ions and this is associated with the sliding plane i.e., the imaginary plane that separates immobile ions on the surface from mobile ions. In most cases, the Stern layer and the sliding plane are close to each other [40].

Ψδ can be approximated with the ZP [41]. Thus, the zeta potential is the difference between the electrical potential on the shear surface of the particle and the electrical potential of the solution, which can be determined by assessing the velocity of charged species moving towards the electrode, in the presence of an external electric field through the sample solution. According to the three-layer model, the variation of the potential as a function of the distance shows first a linear decrease through the stern layer and then a second linear decrease until the sliding plane. Finally, it shows an exponential decay related to the ZP [41].

The zeta potential measurements provide an accurate analysis of the electronic state of the surface of the nanoparticles, and the data obtained can be used to predict their stability. The instability can result from the interaction between nanoparticles with either excess or insufficient load, leading to the formation of aggregates. The ZP with a value of ±30 mV and is generally chosen to infer the stability of the particles, whereby an absolute value higher than ±30 mV indicates a stable condition, while a low ZP value less than ±30 mV indicates a condition instability (favoring aggregation, coagulation, and flocculation) [23]. Notably, a cost-effective polymeric device has been fabricated recently, that allows size and surface charge characterization of different nanoparticles in salt gradient [42].

Light scattering techniques, such as Electrophoresis Light Scattering (ELS), are currently used to determine the zeta potential. It can simultaneously measure the velocities of many charged particles loaded in liquid. Despite being widely used, there is the possibility of decreasing precision and reproducibility of the technique due to electro-osmotic effects [43,44]. Although measuring the ZP of particles in suspension after dilution reduces the difficulty of penetrating light into the sample solution, it is important to note that the zeta potential is a property sensitive to environmental changes, including pH and ionic strength [45].

### 2.5. Scanning Electron Microscopy (SEM)

The scanning electron microscope generates images through the interaction of an electron beam with the sample surface, which is then reflected, using the vacuum as a propagation medium [41]. A three-dimensional image is generated. There are two important phenomena during the emission of electrons: the process of elastic spreading and the process of non-elastic spreading, in which the difference is the conservation of energy by the electrons [25]. This entire procedure can be applied to nanomaterials and sample preparation is a simple procedure. 

### 2.6. Transmission Electron Microscopy (TEM)

A transmission electron microscope is widely used for the characterization of nanomaterials and the image is formed due to the passage of electrons through the sample, allowing us to observe the internal structure [41]. Unlike SEM, sample preparation is complex and time-consuming, which is a disadvantage. One can, however, gain detailed information that is unavailable or limited with the SEM technique, such as complex morphologies or size distribution of the nanoparticle system. The propagation medium is the vacuum and the image appears in a light or dark field [46].

TEM has a spatial resolution power superior to SEM due to the use of electron beams with high voltage, which may destroy the sample [25]. Another disadvantage of this method is the necessary use of a fine sample so that the electrons can pass through it, usually with a thickness less than 50 nm. This method is widely used for the analysis and study of drug-loaded nanoparticles since it allows the detection of the structure of the nanoparticles after the integration of an active ingredient [46].

### 2.7. Scanning Probe Microscopies (SPM)

#### 2.7.1. Atomic Force Microscopy (AFM)

Atomic force microscopy (AFM) is a technique that allows obtaining real images, in three dimensions, of the topography of surfaces, with a spatial resolution that approximates atomic dimensions [47]. It does not require measurement of electrically conductive surfaces and is a Scanning Probe Microscopy (SPM) imaging tool consisting of a micro-machined cantilever (usually made of silicon or silicon nitride) with a sharp tip at one end to detect the reflection of the cantilever [48].

In AFM, the sample surface is scanned using a force-sensitive probe, which consists of a tip with atomic dimensions integrated into a moving arm [49]. As the tip approaches the surface, the atoms at the tip interact with the atoms and molecules on the material’s surface, causing the arm to deflect. This deflection of the AFM arm is measured by changing the direction of a laser beam, the reflected laser beam being captured by a slit photodetector. The AFM probe follows the contours of the surface. During the tip’s displacement across the surface, the computer analyzes, at each position on the surface, the interaction force between the AFM tip and the sample and draws a diagram of the heights, building the topography of the molecule [50]. During the sweeping of the sample, the AFM arm maintains the oscillatory movement, touching the sample only once in each oscillation cycle, decreasing the interaction with the surface and minimizing the damage caused to biological samples. The biological molecules must be immobilized on the surface of a very flat solid substrate, with atomic resolution, for example, the highly oriented pyrolytic graphite (HOPG) substrate. However, the DNA molecules interact weakly with the surface of the HOPG substrate and the AFM tip tends to clean the surface, sweeping the adsorbed molecules to the edges of the graphite substrate steps [51].

#### 2.7.2. Scanning Tunneling Microscopy (STM) and Scanning Tunneling Spectroscopy (STS)

**Figure 2:**
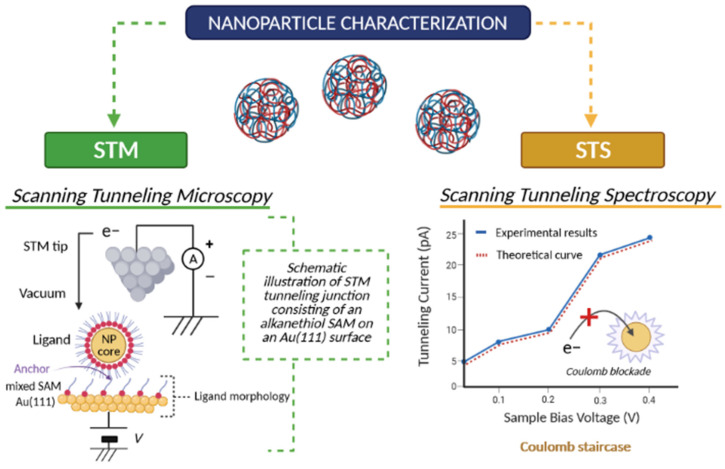
Schematic representation of Scanning Tunneling Microscopy (STM) and Scanning Tunneling Spectroscopy (STS) techniques for the characterization of nanoparticles [own drawing].

Scanning Tunneling Microscopy (STM) represents a technique that allows one to reach an atomic resolution (room temperature: ca. 1 A and ca. 0.01–0.03 A of lateral and vertical resolution, respectively) at flat, conducting surfaces [52,53]. It operates based on the quantum tunneling phenomenon and due to that the local density of electronic states (LDOS) and electronic properties of the nanoparticle system can be established through Scanning Tunneling Spectroscopy (STS) [54]. Changes in the tunneling current that is generated between the STM tip and the surface of the material allow for the investigation of the latter’s morphology, all controlled by the piezoelectric scanner with the angstrom-resolution [55]. These render those techniques to be of choice for studying the applicative potential of various conducting NPs in nanodevices and electronic applications. The latter has been reviewed by Majima and co-workers (2015) [56], highlighting novel functions related to the nature of conductive NPs. Both STM and STS techniques used for the characterization of NPs are shown in Figure 2.

Applications of biological materials are somewhat limited by the poor electron conductivity of most biological samples [57], nonetheless, recent advances allowed e.g., the determination of the spatial structure of polysaccharides using STM technique [58].

### 2.8. Porosimetry

Porosimetry is a method used for the characterization of porous materials and allows the determination of the surface area, volume and pore size of a given sample. The pores are categorized into groups according to their dimensions, namely, macropores (if larger than 50 nm), mesopores (if between 5 nm and 50 nm), and micropores (if smaller than 2 nm) [59].

Porosimetry is widely used in the pharmaceutical area, for example, to determine the life span usefulness of a drug, its bioavailability as well as its rate of dissolution. Recently, it has been of particular importance within the field of various 3D porous materials, such as Metal-Organic-Frameworks (MOFs) and Covalent Organic Frameworks (COFs) [60].

### 2.9. Differential Scanning Calorimetry (DSC) and Thermogravimetric Analysis (TGA)

Thermal analyses such as Differential Scanning Calorimetry (DSC) and Thermogravimetric Analysis (TGA) are very often successfully employed for the characterization of nanomaterials of different compositions [61,62,63]. Calorimetry assesses a sample through changes in enthalpy, that is, through temperature. Temperature changes involve energy release or absorption that give rise to endothermic and exothermic signals. An example is a transition from the crystalline phase to the amorphous phase, which requires high energy absorption. However, processes that involve small energy differences, so it is necessary to ensure that the device has a high sensitivity [29]. In the pharmaceutical area, it is widely used to determine the degree of purity, the change of form of the compounds with the change in temperature, the stability of the sample as well as its melting point [64]. In TGA, the material is heated at a specific temperature, followed by monitoring of the mass changes. Consequently, the mass of the studied sample will increase or decrease, providing information on the physicochemical changes in the structure and/or composition of the compound under study. Most often, one would observe desorption or vaporization of small molecules (water, solvent, etc.) or oxidation/decomposition, depending on the inherent properties of the compound and the combusting atmosphere (oxygen, inert gas, etc.) [65]. In combination with DSC or other techniques such as infrared spectroscopy, mass spectrometry, or gas chromatography-mass spectrometry a comprehensive evaluation of the thermal characteristic of nanomaterials is possible [66].

### 2.10. Fluorescence Spectroscopy

Fluorescence correlation spectroscopy provides images based on the fluorescent light emitted by a fluorophore. It encompasses changes in fluorescence intensity that occur over time. This method uses nanomolar sample concentrations or lower, so it is a very sensitive technique and through this, we can obtain information on diffusion coefficient, hydrodynamic radius, concentration, dynamics of singlet-triplet states, and kinetic chemical reaction rate [67].

It is being widely applied in the area of bioassays and biophysics, particularly in vivo applications, due to its limited invasiveness. To overcome limitations of the technique such as high signal-to-noise ratios and longtime traces, an alternative framework that shortens the latter one has been designed recently [68].

### 2.11. Raman and Infrared Spectroscopy

Raman and infrared spectroscopies are widely used methods for the structural characterization of nanoparticles and use the interaction of light with matter to determine the composition of a material [69]. The information provided by Raman spectroscopy is the result of a light diffusion process, while infra-red (IR) spectroscopy is done by absorbing light. The obtained spectra, which are typical of a given substance, are important for its identification of a substance [70]. The principle of Raman’s spectroscopy is to measure the inelastic dispersion of photons that have different frequencies than incident light after interacting with the molecule’s electrical dipoles [71]. This process then results from frequency differences between incident photons and scattered photons inelastically associated with the characteristics of molecular vibrational states. It will thus investigate changes in the polarizability of molecular bonds. Inelastically dispersed photons emit lower frequencies than incident photons. Thus, there are Stokes lines and anti-Stokes lines [72]. Molecular bonds have specific energy transitions in which a change in polarizability occurs, giving rise to the active modes in Raman. Raman spectroscopy is generally considered complementary to infrared spectroscopy, that is, the vibrational modes active by Raman must be inactive by infrared and vice versa, for small symmetrical molecules, because Raman’s transitions result from the nuclear movement that modulates the polarization of the molecules, rather than the net change in the dipole moment of the molecules [72]. One of the main advantages is being suitable for the study of biological samples in aqueous solution because the water molecules tend to be weak Raman dispersers, in addition, the detailed molecular information offered by this type of spectroscopy can be used to investigate conformations and concentrations of tissue constituents. While it has the advantages of providing an indirect characterization of nanomaterials, such as average size and size distribution through the analysis of the widening and displacement of the spectral line, it “fails” with regard to the spatial resolution necessary to outline different domains for application in nanotechnology. Other disadvantages are fluorescence interference and extremely small crossings, requiring intense laser excitation and a large number of samples to provide sufficient signals [73].

Electromagnetic radiation in the infrared (IR) frequency range is absorbed by molecules, and this promotes vibrational transitions, associated with stretching and twisting connections [73]. Stretch or axial deformations occur when the deformation occurs in the direction of the molecule’s axis causing the interatomic distance to alternately increase and decrease. As for torsional or angular deformations, a group of atoms moves in relation to the rest of the molecule, without changing the positions of the atoms in the group. The IR spectrum thus allows us to study the structure of several molecules. Fourier Transform Infrared (FTIR) and Attenuated Total Reflection Fourier Transform Infrared (ATR-FTIR) spectroscopies are widely used today and one of their great advantages is that they can be applied to nanoparticles. The second method allows the study of the sample’s surface composition, as well as all changes that occur in it [74].

### 2.12. Circular Dichroism (CD)

Circular dichroism is a spectrophotometric method applied for optically active systems, based on differentiated absorption of clockwise and counterclockwise polarized light. The light absorbed in each of the directions is different, and this technique measures, precisely, that difference in absorption between the circularly polarized light on the right (RCP) and the left (LCP) through chromophores in optically active chiral substances [75]. In linearly polarized light, the electromagnetic wave oscillates in only one plane, whereas in circularly polarized light it occurs in two planes [76]. When overlapping, RCP and LCP, originate a linearly polarized wave. An overview of the technique within the pharmaceutical applications of NPs has been given recently [77].

Spaeth et al. (2019) [78] have recently introduced the concept of photothermal circular dichroism, allowing one to achieve a superior signal-to-noise ratio for the detection of chiral nano-object. The chiral response of single plasmonic nanostructures with CD in the visible range was demonstrated for the first time, extending CD studies to individual NPs and significantly improving the conventional CD spectroscopy.

### 2.13. Nuclear Magnetic Resonance (NMR)

Nuclear magnetic resonance is used to identify the connectivity of atoms in a molecule [65]. The sample is placed in a constant magnetic field, is irradiated with a short radiofrequency pulse that excites all nuclei in different environments, at once. The proton nuclei (^1^H), carbon-13 (^13^C), and some other elements and isotopes behave like small magnets, which when placed in an external magnetic field and irradiated with energy in the radio frequency range, absorb energy at frequencies modulated by its chemical environment [79]. The proton spin represents the possible orientations that charged particles and some nuclei can present when subjected to a magnetic field and acts as a magnetic bar. The common hydrogen nucleus is like an electron, its spin is ½ and can assume two states: + ½ and -½, which means that the hydrogen nucleus has two magnetic moments [80]. Nuclei that do not have a spin, such as ^12^C, do not respond to an external magnetic field. It can be used as a nanoparticle characterization technique since it allows determining the structure, size, purity, functionality, and conformational changes resulting from the interactions that occur between them. NMR is a non-invasive technique with reduced sensitivity, which implies the use of a large amount of sample [81]. Over time, the “high-resolution magic angle spinning (HR-MAS) NMR” method has been adopted, a technique with great potential for the characterization of living tissue samples. One of the advantages of this method over conventional NMR is that after obtaining the spectrum, the tissue sample remains intact [80].

### 2.14. Mass Spectrometry (MS)

Mass spectrometry involves the production and detection of entities of different mass to charge ratios, resulting in an ionic mass-ionic abundance spectrum. It is a method used to identify and quantify compounds in a complex organic mixture, identify structures of biomolecules, determine how drugs are used by the body, among others [82]. It has a high specificity as it can measure molecular masses accurately and provide structural information from ion fragments. It can be applied to all types of samples regardless of the state, polarity, or volatility. It has also high sensitivity. This method is characterized by two steps [83]:

(a) Ionization—the molecule is bombarded by a high-energy electron beam,
(b) Fragmentation—occurs when excess vibrational energy is transferred to the molecular ion, with a process of scission of the bonds that hold the molecule together causing fragmentation. This technique can be used to determine the molecular weight, the atomic composition and the structural blocks observed through fragmentation [83].

### 2.15. Rheology

Rheology, derived from the Greek word “Rheo” means flow, is the science that studies deformation and the movement of matter and is directly related to viscosity. All fluids have some viscosity, that is, they offer resistance to movement, applying tangential forces to surfaces in contact with it, thus counteracting movement. Viscosity is directly proportional to the size and polarity of the molecules. Another important factor for the study of viscosity is the temperature that is directly proportional to the viscosity in liquids and inversely proportional in gaseous fluids. Fluids can also be divided into two major groups: Newtonian and non-Newtonian fluids. The difference lies in the fact that the former has a linear relationship between the shear stress and the strain rate, and the latter a non-linear relationship [84].

## 3. Effect of Physicochemical Properties on Nanopharmaceuticals Performance

### 3.1. Size

**Figure 3:**
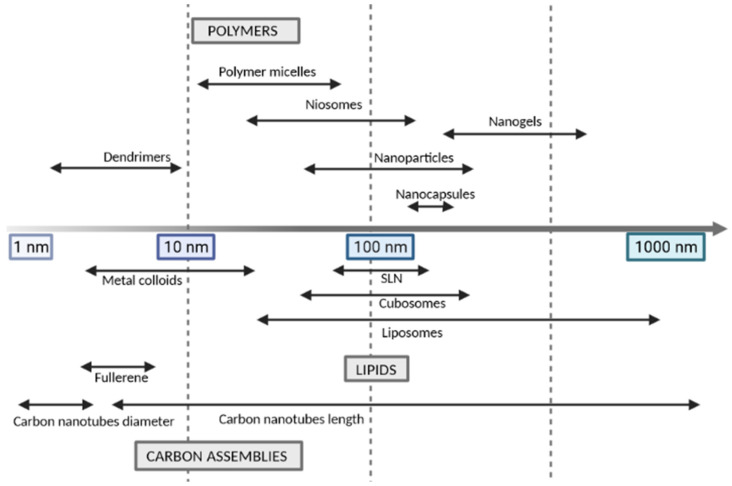
Graphical representation of different types of nanomaterials and their corresponding size range [own drawing].

The most effective nanopharmaceuticals should have a size between 1–100 nm [85], although nanoparticles’ size may generally range from 1 to 1000 nm, as shown in Figure 3. This physical property interferes with the delivery of the active ingredient and its bioavailability, at various levels. This type of structure has numerous advantages, such as protection against its degradation in a physiological environment, controlled release of the active principle via targeted manner, in addition to allowing the administration of hydrophobic drugs in aqueous media [86]. It can reduce possible side effects, they also influence the absorption, distribution, metabolism, and elimination of the drug. However, we must take into account the risk of nanotoxicity, commonly associated not only with the type of nanoparticle but also with their size and size distribution [87].

### 3.2. Surface Properties

Neutral or negatively charged, and hydrophilic particles tend to promote permeability and retention. They are usually coated with a “stealthing agent”, such as polyethylene glycol (PEG) [88,89]. PEG has adequate resistance to adsorption by proteins and subsequent phagocytic activity. The density of PEG needed to promote resistance to proteins and blood circulation varies greatly with the type of nanodrug. Normally, immunological or metallic particles require a larger PEG surface compared to other more inert particles. To deliver the drug well-targeted, the nanoparticles can be coated with specific ligands for certain receptors on the affected cells [89]. One has to bear in mind that although PEGs are FDA approved, prolonged contact through consumption or drug administration can lead to the formation of the anti-PEG antibodies, resulting in sometimes severe, allergy-based immune system responses [90]. A series of alternative, PEG-related compounds was recently reviewed [90,91] e.g., poly-(acrylamides)/(carbonates)/(amino acids)/(glycerols)/(*N*-vinylpyrrolidone) or zwitterionic polymers, nonetheless, the PEG-immunization problem should be considered serious [92].

### 3.3. Passive versus Active Targeting

**Figure 4:**
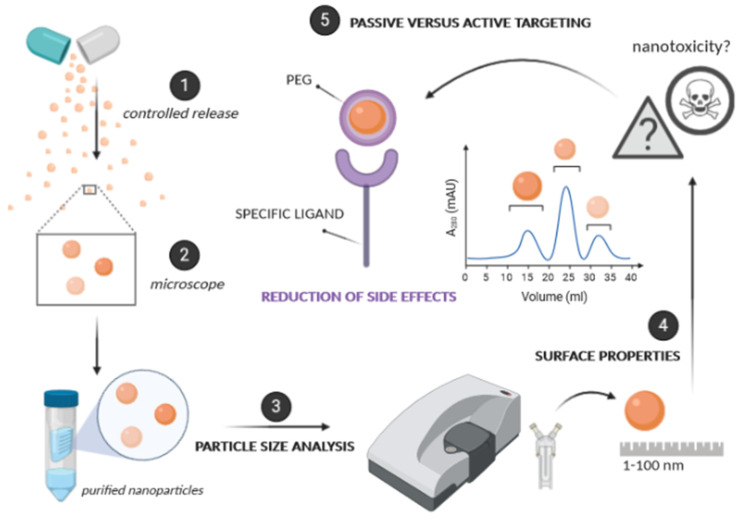
Graphical summary of the most important features of nanoparticles towards their successful implementation for healthcare applications [own drawing]. Explanations of the key stages of the implementation process for nanoparticles: 1—controlled drug release; 2—microscope analysis (SEM, TEM, SPM); 3—particle size analysis (DLS, SLS, ZP); 4—assessment of the surface properties; 5—evaluation of passive/active drug targeting.

The ability of nanoparticles to focus on the action site is achieved by balancing passive versus active targeting [93,94]. In passive targeting, the successful delivery of the drug is directly related to the circulation time of the particles [95]. This is achieved by wrapping the nanoparticles with a type of coating. As mentioned above, many are coated with PEG. By adding PEG to the surface of the nanoparticle, they become hydrophilic [96]. This process makes the substance antiphagocytic, due to natural hydrophobic interactions of the endoplasmic reticulum. The active targeting is based on improving the effects of passive targeting, making the nanodrug specific to a site of action [97]. This active targeting can be achieved by knowing the nature of the target cell’s receptors, and then the specific ligands that allow the connection to its complementary receptor can be used [98]. After ligation, mediated endocytosis may occur. Most of the body has a consistent pH, but there are areas more acidic than others, and we can take advantage of this parameter; another feature is the redox potential. If we combine passive and active targeting, the nanodrug has a great advantage over conventional drugs [95]. Although there are so many advantages, such as the reduction of side effects, the area of nanotoxicology is a growing field of study that is concerned with harmful effects on health and the environment [99]. The most important features of nanoparticles towards their successful implementation for healthcare applications are shown in Figure 4.

## 4. Cancer Nanopharmaceuticals

The concept of cancer immunotherapy is based on the use of the host immune system to control the growth of tumors. It has great potential to treat and prevent the recurrence of cancer [100]. Demonstrated successes in clinical trials are not only a motivation for further studies but also a bridge towards the real-life application of these methods for health care [101]. Despite that, however, most challenges that remain to be solved revolve around the suitable delivery methods, combined with conventional treatment as well as prediction and online evaluation of the triggered immune responses [102]. To overcome those, one needs to understand the cancer immunity cycle, so that the death-induced release of the cancer cell antigens does not harm the normal cells.

**Figure 5:**
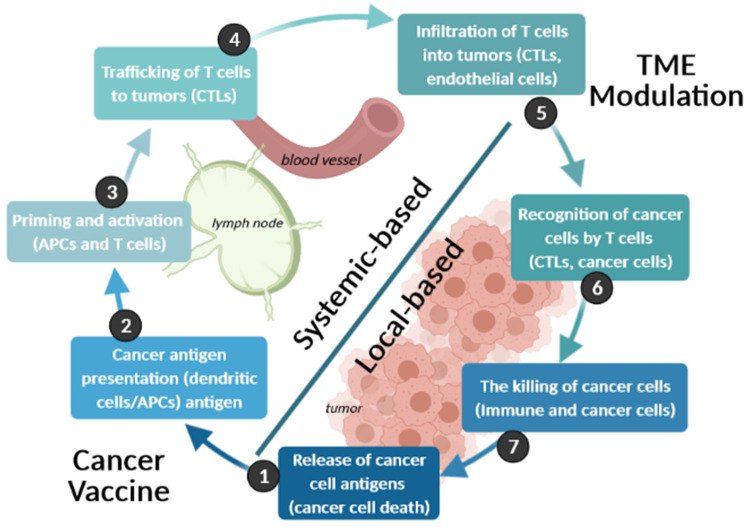
Schematic representation of the cancer-cell immunity cycle [own drawing]. Explanations of the abbreviations: APC—Adenomatous Polyposis Coli; T cells—thymus cells; CTLs—cytotoxic T lymphocytes; TME—tumor microenvironment.

The overall process starts with the release of the cancer cell neoantigens and is finished upon the death of the cancerous cell, which is summarized above (Figure 5) and is divided into 7 stages as detailed by Chen and Mellman [103]. At the start, either neoantigens may be presented to the immune system by the cancer cell themselves via Major Histocompatibility Complex class I (MHC-I), or release of the neoantigens may occur via Immune system independent tumor cell death or even in the absence of tumor cell death (1). Released neoantigen is captured by the tissue antigen-presenting dendritic cells (APCs). These cells then become activated, and while processing the antigen, migrate via lymphatic circulation to regional lymph nodes where they presented the processed antigen to T-cells via MHC-II (2) leading to neoantigen-specific T cell activation (3). Activated T-cells migrate from lymph nodes via the blood circulation (4) and recognize inflammatory response modified endothelial cells in the tumor tissue (5) adhere and permeate it. Specific MHC bound tumor neoantigen peptides are then recognized by the unique T-cell-Receptor (TCR) [104] of these anti-tumor cells (6) allowing for tumor cell-specific killing (6) which propagates the cycle as phagocytic dendritic cells and/or macrophages take up the released cancer cell antigens in the process of clearing apoptotic cell debris. The response is thus amplified at each cycle [105].

For effective cancer therapy, it is necessary to improve and develop new strategies for the effective delivery of chemotherapy to cancer cells [106]. Conventional chemotherapeutic agents accumulate in both normal and tumor cells, due to non-specificity. The ultimate goal of cancer therapy is to reduce systemic toxicity and improve quality of life [107]. As such, we now describe four methods of altering different steps of the cancer-cell immunity cycle, with the improved resulting nanotherapeutic outcomes.

### 4.1. Ligand/Receptor Targeting

Ligand/receptor targeting has proven to be an effective method for drug delivery [108,109]. To improve effectiveness, chemotherapeutic agents need to be administered into the cytoplasm of cellular tumor cells, or into subcellular organelles such as the nucleus and mitochondria [110]. If a linker is involved, a stable covalent bond formation is essential between the linker/receptor and the drug. However, the mechanism of drug release at the tumor site is crucial [111]. The premature release can result in systemic toxicity. The tumor’s target ligands are being explored to target metastatic cells and block their migration and invasion [112].

Drug delivery can be classified as active and passive [93]: passive aiming explores the systemic and lymphatic systems in tumor architecture, which is known as the enhanced permeability and retention (EPR) effect [113]. The effect of nanoparticle size, charge, and PEGylation on the EPR has been already reviewed [114]. The active target includes ligand-mediated drug delivery. These ligands can be conjugated covalently with an active agent or on the surface of a carrier system, such as nanoparticles, liposomes, or nanomicelles [115]. These ligand groups can recognize certain surface molecules that are overexpressed by cancer cells, contrary to the normal cells, where they are absent [116]. There are several targeting strategies for administering anticancer drugs [117], (some of) the most promising/widely studied approaches are described below.

#### 4.1.1. Small Molecule Receptors for Lectin and Foliates

Carbohydrates were recognized to be effective agents to interact with lectin membrane proteins, thus triggering lectin-mediated endocytosis. Expression of Galectin-3, a galactose-binding lectin, correlates with the proliferation of colon cancer cells, and therefore utilization of therapeutics with appended-sugar moieties can be envisaged [118,119]. Indeed, Rubinstein and co-workers demonstrated that from the studied copolymer conjugates of *N*-(2-hydroxypropyl)methacrylamide with appended lactose and galactosamine sugars, the latter one exhibited significant binding towards the colon adenocarcinoma [120]. Recently, a series of bottlebrush glycopolymers were synthesized via the Cu-mediated atom transfer radical polymerization (ATRP) process, with mannose-moiety appended through the *grafting-from* methodology, which could find applications as drug-encapsulation and delivery systems with strong lectin-binding features [121].

Another important receptor associated with the cancerous activity is the targeting of the folic acid receptor, which is overexpressed in some cancer cells [122,123,124,125]. The folate receptor has been considered of particular importance for ovarian and lung cancer and utilization of NPs within this domain was reviewed some time ago [126]. Tatsuya et al. (2017) described the preclinical models of porphysomes (porphyrin-lipid nanoparticles) targeted to folate receptor 1 (FOLR1) for enhancement of the efficiency and specificity of photodynamic therapy (PDT) [127]. The intracellular uptake of NPs was observed in vitro and corresponded with the expression of folate receptors in lung cancer cell lines.

#### 4.1.2. Drug Antibody Conjugates

These systems are highly specific and are used as decorative moieties onto various NP systems for targeting transmembrane glycoproteins named epidermal growth factor receptors (EGFR) [128]. Some antibody conjugates have been marketed for cancer nanotherapeutics, such as brentuximab vedotin (Adcetris^®^) and emtansine trastuzumab (Kadcyla^®^). The latter one as well as cetuximab can target cancer cells with EGFR and tyrosine kinase receptor (HER-2), which are overexpressed in cancer cells, particularly in breast tumors [7,129]. Currently, most antibody conjugates are in different stages of clinical trials but some are still in the early stages of development [130].

#### 4.1.3. Aptamers

These macromolecules are isolated from DNA or RNA that bind to proteins and peptides with high specificity and affinity [131]. Aptamers recognize several targets ranging from small molecules to macromolecules, so they are used in therapeutic and diagnostic applications [132,133]. They are highly resilient towards tissue and blood nucleases and can be readily functionalized at the carbohydrate backbone or free 3′amino-groups to enhance their efficacy or influence other biological characteristics [134]. Aptamer-based supramolecular nanotheragnostics have been reviewer recently, with effective aptamer–receptor binding attributed to a combination of non-covalent, supramolecular interactions (e.g., H-bonding, π–π interactions, and van der Waals forces) [135]. Several studies have shown that they minimize systemic toxicity and increase drug release at the tumor site [136]. As for 2016, there were three aptamers used in ophthalmology, including one FDA-approved (US Food and Drug Administration) drug pegaptanib (Macugen), and two in late-stage development (ACR-1905 and E-10030) [137,138].

#### 4.1.4. siRNA

The surface of calcium carbonate nanoparticles coated with siRNA was shown effective in the suppression of vascular endothelial growth factor C (VEGF-C) in gastric tumors [139,140]. Likewise, the surface-modified nanoparticles generated greater transfection efficiency in the human gastric cell line (SGC-7901) compared to the blank non-conjugated nanoparticles [141].

In vivo studies suggest that calcium carbonate nanoparticles combined with siRNA may inhibit the growth of cancer cells [139]. On the other hand, targeted administration of anti CD47 siRNA conjugated to liposomal protamine hyaluronic nanoparticles (LPH-NPs) to lung cancer cells significantly inhibited cancer metastasis (~27%), suggesting that administration of active siRNA is highly effective [142].

#### 4.1.5. Peptides 

Peptides enhance the anti-angiogenic effect and can be targeted as anti-angiogenic agents for tumors [143]. A phage display peptide library series has been screened in general to identify peptides with a high affinity for cancer cells [144]. The improvement of the EPR effect due to hypervascularity, poorly differentiated vasculature, and ineffective lymphatic drainage is the main responsible for the development of weak, fragile, and leaking vasculature [145]. Such a passive target explores the systemic and lymphatic systems in the tumor architecture. Certain aggressive tumors can develop a pore from 100 to 800 nm due to neovascularization [146]. Nanometer-sized drug carriers can take advantage of these pores and accumulate at the tumor site due to the EPR effect. There are reports that small particles (20 nm–100 nm) with superficial pegylation can prolong circulation. Such carrier properties can assist in a greater accumulation of particles at the tumor site and increase diffusion within the target tissues [89,96,147].

#### 4.1.6. Cell-Penetrating Peptide (CPP) and Transferrin (Tf)

CPP can serve as an effective ligand for cancer therapy. CPPs are generally composed of 5 to 30 amino acids, basic or amphiphilic, and efficiently translocate the plasma membrane, and can assist in the translocation of drugs across the cell membrane [148].

Transferrin (Tf), a protein that regulates the iron uptake, can also be a targeting molecule for therapeutic genes and/or drugs related to the treatment and diagnosis of cancer [13,149,150]. The binding of Tf with nanoparticles resulted in systems that target transferrin receptors in cancerous cells and enable nanotherapeutics with enhanced selectivity and safety. Clark and Davis [151] have shown how Tf-appended NPs can effectively cross the blood-brain barrier and release the therapeutic nanoparticles in the brain, hitherto restricted by the endothelium. Tf coupled with copper nanoclusters and doxorubicin (DOX) was exploited for bioimaging and target drug delivery [152]. Such formed nanoparticles were evaluated in vivo and confirmed pronounced inhibition of tumor growth in mice using the Dalton Lymphoma ascites (DLA) model. Soe et al. (2019) demonstrated on the other hand how one can improve the DOX delivery via utilization of the polymeric nanoparticles for the Doxorubicin-Resistant breast cancer cells [153].

### 4.2. Intracellular Targeting

The most effective technique for targeting tumor cells is to target DNA-inhibiting drug molecules to cancer cell nuclei [146]. The nuclear target not only mainly causes tumor cell death, but also minimizes damage to surrounding normal cells [154]. The main problem with such targeting is to avoid translocation of active agents into endosomal or lysosomal vesicles [155,156]. The drug delivery mechanism requires active molecules to escape subcellular cytoplasmic vesicles and translocate them to nuclei. Cancer cells develop mechanisms of intracellular resistance, such as overexpression of drug efflux pumps, metabolism, and sequestration in acidic compartments, and deactivation [157]. There are two strategies for drug transport: direct nuclear targeting (drug molecules are transported to the cytosol in large quantities, subsequently allowing the capture of nuclear DNA) and indirect nuclear targeting (nanotransporters transport molecules to cancer cells through the cell membrane to the cytosol and finally are located in the nuclei where the active molecules can be released) [158].

The main barrier to the supply of nuclear drugs to mammalian cells is the plasma membrane that restricts the passage of large and charged hydrophilic molecules, causing the large nanotransporters to be carried by different endocytotic mechanisms into the cell [159]. Nuclear membrane and nuclear transport are other selective barriers in eukaryotes. The nuclear envelope involves the nucleus and separates the nucleoplasm and the genetic material from the cytoplasm. It includes nuclear pore complexes (NPC) through which the exchange of molecules occurs [160,161]. Each individual NPC translates approximately 1000 proteins per second in a bidirectional manner. Large molecules are transported via target signals to enter or leave the nucleus, on the other hand, small molecules pass through the NPC by passive diffusion [144,160].

Four major targeted nuclear delivery systems have been studied extensively: (i) NLS-mediated delivery system; (ii) TAT, a combined nuclear delivery system; (iii) Delivery system based on cationic polymers; (iv) Load inversion approach with triggered pH. In comparison with conventional chemotherapy that can cause high toxicity due to lack of specificity, gene therapy offers a unique and powerful approach to fight cancer [162]. It works at a molecular level, in which genetic materials or functional genes are inserted into patients’ cells to repair or replace defective genes [163].

Gene delivery methods were developed for gene therapy, which can generally be divided into two categories, viral and non-viral delivery systems [162,164].

**Viral vector:** Viruses are microscopic infectious agents that can replicate in living cells. Researchers have used viruses to deliver therapeutic genes to cell nuclei due to their high transfection efficiency, ability to penetrate, express, and replicate in host cells. To use the virus as a vector, it is necessary to remove the pathogenic part of the viral genes and replace them with therapeutic genes. The remaining non-pathogenic segments of the virus carry the therapeutic gene that constitutes the viral vector [165].

**Non-viral:** Many non-viral systems have been investigated for gene delivery, including DNA injection or physical methods, such as electroporation [164].

### 4.3. Immunotherapy

Immunotherapy is a type of biological therapy, which it involves increasing the effectiveness of the human immune system to prevent the proliferation of tumor cells or their elimination [166]. There are 3 main types of immunotherapy for the treatment of cancer, i.e., non-specific immune stimulation, T-cell transfer therapy, and checkpoint inhibitory therapy [167]. Non-specific immune stimulation occurs by stimulating non-specific immune mechanisms of action. These mechanisms are physical barriers (e.g., skin), chemical barriers (e.g., stomach acidic pH), complement system (biochemical cascade that attacks the surface of invading cells), cellular barriers (e.g., macrophages), and increased production of interleukins or interferons [168]. T-lymphocyte transfer therapy is based on obtaining T lymphocytes from the patient, which are cloned in vitro, and transferred back to the patient [169]. In the T lymphocyte, a gene is inserted that encodes a receptor, which recognizes a specific antigen of the tumor cells. These T lymphocytes bind to tumor cells and destroyed them [170]. Checkpoint inhibitor therapy, which is a fundamental regulator of the immune system, can be used to sustain an immune response. Some types of tumors can protect themselves from the host’s immune system by stimulating these checkpoints [171]. In this therapy, the inhibitors of these checkpoints act, restoring the immune system to function properly. Examples of inhibitors: CTLA-4; PD-1 and PD-L1. PD-L1 is the ligand for the programmed death transmembrane protein PD-1. When PD-L1 (in the tumor cell) binds to PD-1 (in the T lymphocyte), there is inhibition of immunological activity [172]. If antibodies bind, either PD-1 or PD-L1, they block the interaction [173]. Thus, the T lymphocyte is not inhibited and can attack the tumor cell.

### 4.4. Controlled-Release Strategies

The administration of nanotherapeutics is convenient in that it fights breaks in conventional administration. Delivery takes place in specific parts of the body, without affecting healthy tissues mainly by the type of nanomaterial [86]. These nanomaterials are customized in such a way to increase their selectivity and accumulation in tumors by the EPR effect [113,174]. There are two distinct groups of nanomaterials, inorganic and organic.

#### 4.4.1. Inorganic Nanomaterials

Quantum Dots (QDs) work as probes that allow obtaining complex and long-term images, and diagnostics [175]. They are fluorescent 0-D (Zero Dimension) nanoparticles used in drug delivery and allow in vivo monitoring. It has disadvantages such as the fact that they are hydrophobic and tend to aggregate, so it has to be coated with a layer of ligands so that they become soluble [176].

Carbon Nanotubes (CNTs) are 1-D (One Dimension) nanomaterials that increase the temperature of the tumor making it noticeable by methods of Infrared radiation and thermal photography [177]. Biological membranes pass easily and transport molecules to the cytoplasm without toxic effects [178].

Layered Double Hydroxides (LDHs) can exchange anions, it is low cost and easy to prepare, easily penetrates cell membranes, and is easily expelled by endosomes. They consist of layers of divalent metal ions with a substituted trivalent metal to provide positive charges [179].

Mesoporous silica nanoparticles (MSNs) have recognized excellent structural properties, among which high surface area, large pore volume, narrow pore size distribution, or tunable pore diameter can be highlighted. Based on the silica and surfactant content, several forms of MSNs can be produced, including the most common types, such as Mobil Composition of Matter No. 41 (MCM-41) and Santa Barbara Amorphous-15 (SBA-15) [180].

#### 4.4.2. Organic Nanomaterials

Polymeric nanoparticles (colloidal and biodegradable solids) are easy to produce and modify structurally, such as changing surface properties. The drug can be encapsulated by dispersion in the polymeric matrix [12,181].

Liposomes are small and spherical structures with at least one lipid barrier with an aqueous phase inside. They are biodegradable, can encapsulate hydrophilic substances (example: hydrophilic drugs; DNA; RNA), and act passively or actively. The main associated problem is its short duration in the bloodstream [182]. One however has to note that recently devised and globally administered BioNTech/Pfizer’s and Moderna’s mRNA COVID-19 vaccines utilize lipid nanoparticles as mRNA carriers, demonstrating their utility [183].

Protein nanocarriers (e.g., albumin) can be obtained from several sources. It is not immunogenic, has a high capacity to bind to various drugs (due to the amine and carboxylic group on its surface), and has a long life in the circulating plasma [184].

Micelles are spherical or globular colloidal complexes. They are hydrophobic, with a hydrophilic outer layer. They can be formed spontaneously under certain conditions of concentration and temperature [185].

**Figure 6:**
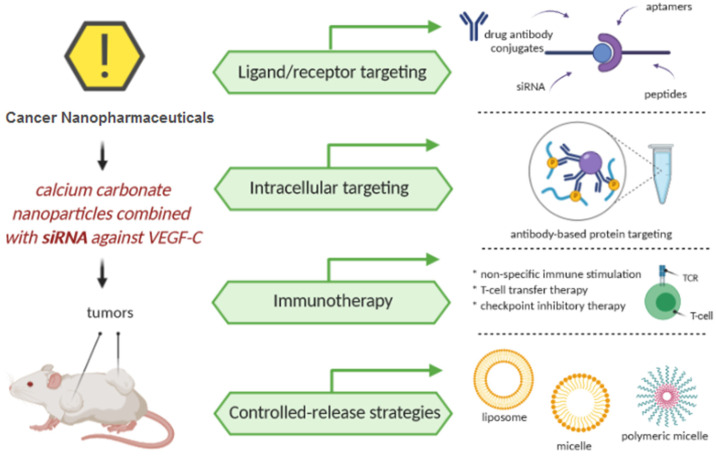
Graphical summary of the chosen methods towards development of the efficient and safe cancer nanotherapeutics [own drawing].

All of the described methods towards development of the efficient and safe cancer nanotherapeutics are schematically shown in Figure 6.

## 5. Nanopharmaceuticals-Based Cancer Treatments

The therapy of malignancies is a dynamically developing area of pharmacology. On the one hand, the novel therapeutic molecules or pharmaceutical formulations are designed to be the most specific and precisely directed against neoplastic cells [186]. On the other hand, these modern anti-cancer drugs should not interfere with healthy tissues to significantly decrease the risk of adverse events. These are the main rules of personalized medicine in oncology [187].

Nanopharmaceuticals of already known or new therapeutic molecules used for oncological patients’ therapy represent one of the newest solutions fulfilling the criteria of personalized medicine. These formulations include for example liposomes, dendrimers, polymeric micelles, gold nanoparticles, iron oxide nanoparticles, artificial exosomes, nanobubbles, silica-based systems, and lipid nanoparticles [188,189].

One of the best examples of how nanoformulations of anti-cancer drugs can improve therapeutic outcomes in hepatocellular carcinoma (HCC). Unfortunately, a significant proportion of patients with HCC is diagnosed in an advanced stage of the disease [190]. One of the most frequently recommended therapeutic options in these cases is transcatheter arterial chemoembolization (TACE) [190]. Different anti-cancer drugs are being used in TACE chemotherapy. Doxorubicin has been shown to be the most effective molecule in HCC by inhibiting DNA replication of malignant cells [191]. The main limitations of doxorubicin use are its toxicity and interactions with the mononuclear macrophage system, which recognizes the drug and interferes with its pharmacokinetics [191]. To overcome these obstacles, various forms of PEGylated liposomal doxorubicin have been developed. In a recent paper by Liao et al. (2020), it has been shown that TACE with raltitrexed plus liposomal doxorubicin reduced the incidence of adverse events and significantly improves the overall survival among patients with unresectable HCC [191]. To improve the specific binding of the drug to hepatocellular neoplastic cells, various approaches have been proposed, but their use was mainly assessed in vitro studies and animal models. Lactoferrin binds specifically to asialoglycoprotein receptors on HCC cells [192]. Wei et al. (2015) showed that doxorubicin-loaded and lactoferrin-modified PEGylated liposomes loaded with doxorubicin had significantly stronger anti-neoplastic properties. This was hypothetically due to enhanced cellular uptake of new drug formula when compared with PEGylated liposomes. Li et al. (2020) presented in their study that dual-ligand-modified liposomes can improve the delivery of doxorubicin to liver cancer cells [193]. In another study, performed by Wang and colleagues, the authors constructed a novel form of doxorubicin-loaded immunoliposomes [194]. The rationale for that is a high expression of HAb18G/CD147 on the surface of HCC cells, which is believed to be a molecular marker of disease progression. Thus, F(ab’)2 of a CD147 was conjugated to PEGylated liposomal doxorubicin. The authors were able to demonstrate that this novel nanoformulation of doxorubicin is highly effective in influencing malignant cells. What is more, it significantly decreased the number of CD133-positive HCC cells, which are believed to play a role in HCC stem cells. The superiority of anti-CD147-conjugated PEGylated liposomal doxorubicin was confirmed both in HCC cells and patient-derived HCC xenograft models [194].

These and other modifications of liposomal doxorubicin nanoformulations are still under investigation and hopefully will increase the efficacy and safety of chemotherapy in patients with advanced HCC. 

Available completed or ongoing clinical trials including usage of gene therapy in cancers were shown in Table 1.

## 6. Cancer Nanopharmaceuticals-Based Gene Delivery

Delivering complex molecules to a specific site of action for gene therapy has led to the development of nanoparticle drug delivery systems. Nowadays there is a huge interest in the development of new therapeutics against cancer using different types of nanoparticles including naked nucleic acid-based therapy, targeting micro RNAs, oncolytic virotherapy, suicide gene-based therapy, targeting telomerase, cell-mediated gene therapy, and clustered regularly interspaced short palindromic repeat (CRISPR/Cas9) based therapy approach [195,196]. Although naked DNA plasmid raises measurable levels of antigen-specific immunity and is effective in some preclinical studies; their efficacy in clinical trials was unsatisfactory in creating effective immunity. As a new treatment modality, gene therapy uses nucleic acids such as small interfering RNA (siRNAs), DNA, and oligonucleotides to silence cancer-causing genes, repair mutant genes, or increase the expression of beneficial proteins that can prevent cancer development. the beneficial effects of concurrent chemotherapy and gene therapy have been observed, especially in overcoming multidrug resistance (MDR). The problematic poor stability, lack of tumor selectivity, and rapid clearance from the body may be limited by the use of nanoparticles as carriers for anti-cancer agents due to their controlled drug release and tumor-selective properties [197]. Various strategies of nanoparticles and bioreductive prodrugs are used to overcome the development of resistance to chemotherapeutic agents, followed by invasion and metastasis from environmental hypoxia, which disrupts DNA repair mechanisms and causes genome instability due to increased production of reactive oxygen species [198]. Liposomes, albumin nanoparticles, and polymeric micelles are already used in cancer treatment, while chemotherapy, hyperthermia, radiotherapy, gene or RNA interference (RNAi) therapy, and immunotherapy are under development [199].

Gene-related nanoparticles should be stable and protect nucleic acids during transport through the circulation and reach the target tissue. The next step is the effective absorption of the molecule into the cell through endocytosis, followed by an escape from the endosome and transfer to the nucleus. Then, the transcriptional activity of the introduced genetic material is expected [200]. Genetic and chemical engineering have made it possible to use adenovirus-associated virus or lentivirus in cell-based gene therapy and cancer immunotherapy as well as modified plant viruses in cancer treatment. Exosomes to transfer anti-cancer loads to target tumors, nanodiamonds and graphene are also being studied [199].

Nanoparticles in the form of vesicular cationic lipid-assisted nanoparticles are capable of modifying the acidic environment instead of using it for targeted purposes. In vivo tests in breast cancer models revealed a therapeutic reversal of tumor acidity mediated by RNAi nanoparticles to restore the anti-tumor function of T cells. Multifunctional targeted nanoparticles like gold nanoparticles are prime candidates for thermo-diagnostic applications are among the frequently reported theragnostic nanoparticles used in cancer treatment [198]. By physically entrapping or chemically coupling various therapeutic or imaging agents to nanocarriers, nanotherapeutic agents have enabled increased solubility, targeted delivery, reduced systemic toxicity, and increased therapeutic efficacy in the treatment of cancer. The effectiveness of nanoparticles is related to the regulation of the carrier size, morphology, as well as surface properties, including charge and targeting molecules. Due to the increased permeability and retention (EPR) effect, nanoparticles preferentially accumulate in tumors due to their leaky vascular system and poor lymphatic drainage. Nanocarriers can also be designed as intelligent formulations for the controlled release of the drug in response to various stimuli in the tumor microenvironment, which is to further improve the therapeutic efficacy of nanoforms [201].

Nanoparticles enable the delivery of drugs used in chemotherapy, photodynamic therapy PDT agents, small molecule inhibitors, and therapeutic genes, allow the release of stimulus-responsive drugs [197]. The use of DNA vaccines in cancer therapy and somatic gene therapy is limited by the degradation of the DNA via nucleases, poor delivery to antigen-presenting cells (APCs), and insufficient uptake of DNA plasmids by cells upon injection decreasing immunogenicity. Accordingly, both viral and non-viral vectors have been used to develop delivery systems that provide protection for pDNA to improve the effectiveness of DNA vaccination strategies [195]. Viral gene therapy may be associated with an acute immune response, immunogenicity, and oncogenesis upon the integration of viral components into chromosomal DNA. Cytokine release syndrome may occur and may progress to macrophage activation syndrome [200].

Non-viral vectors have the advantages of low immunogenicity, high delivery capacity, and easy preparation However, effective non-viral delivery systems remain a major concern in clinical gene therapy because the production of intracellular antigens using foreign DNA more closely mimics live infections, and may trigger cell-mediated and antibody-mediated immune responses [202]. Non-viral gene delivery systems may be divided into three main groups, namely, physically mediated methods (microinjection, ultrasound-mediated microbubble, microparticle bombardment, and electroporation), chemical vectors (cationic polymers and cationic liposomes, shell nanoparticles, and polymeric nanoparticles), and biological methods (bacteria and specific mammalian cells). All types of non-viral delivery have limitations including their significant low transfection efficiency and poor transgene expression [195].

Targeted delivery of NPs can increase tumor accumulation and retention, and cellular uptake of siRNA. The effectiveness of the release of siRNA from endosomes via lipids is not high, it is assumed that approx. 70% of internalized siRNA may undergo Niemann–Pick type C1-mediated exocytosis. Delivery to intracellular organelles such as the nucleus, mitochondria, endoplasmic reticulum, and Golgi apparatus is also planned [199].

Systemically injected nanoparticles accumulate in the liver and spleen as a result of clearance through the reticuloendothelial system (RES), which can lead to nonspecific stimulation of immune cells, cytokine storm, and side effects. Administration of lipid nanoparticles (LNPs) loaded with mRNAs encoding cytokines including interleukin (IL) -23, IL-36γ, and OX40L induces potent anti-tumor responses in a wide range of tumors. Specificity can also be achieved through the action of other factors specific to the tumor microenvironment, including low pH, hypoxia, and highly reactive oxygen species [201].

Naked nucleic acid, especially in the form of small interfering RNA (siRNA) has been used in cancer treatment many times showing inhibition of lung cancer growth in mice, without any significant toxicity. Small interfering RNA can be designed to inhibit the expression of any gene but the siRNA therapies have some limitations including poor transport across biological barriers, limited cellular uptake, degradation, and rapid clearance. Nanotechnology helped to overcome many problems, commercialization, and implementation into the clinic [203]. In the case of siRNA delivery to the cytosol, NP endosomal escape is essential. NPs based on cationic lipids, lipid-like materials, and polymers showed promise for siRNA delivery. Most RNAi nanotherapeutic agents in cancer treatment clinical trials consist of liposomes or lipid NPs [199]. The ability of siRNA to silence genes could lead to increased use as a new anti-cancer drug. Binding to biocompatible cationic polymers allows the delivery of siRNA together with chemotherapeutic agents, of a synergistic nature. Binding to ligands makes it possible to target specific cells. Nanoparticles reduce systemic toxicity and improve efficacy. electrostatic interactions with siRNA cationic components ensure high siRNA protection, loading efficiency, and siRNA release into the cytoplasm of target cells. The use of nano-vaccines appears to be a promising strategy [204]. The use of combination therapy based on nanotherapeutics; stimulus-responsive nanotherapeutics targeting acidic pH, hypoxic environment, or nanoparticle-induced hyperthermia are just some of the approaches that are intensively researched in cancer treatment [198]. To limit cytotoxicity to normal cells, nanoparticles containing chemotherapeutic agents are increasingly being used [205,206,207]. Nanoparticles must overcome obstacles, including biological barriers and the tumor microenvironment (TM), before reaching target cells to demonstrate a therapeutic effect. Nanoparticles carrying siRNA interact with the cell membrane of cancer cells and the mechanism of endocytosis is influenced by the size, shape, charge, and surface chemistry of a nanoparticle. Not all nanoparticles may induce efficient escape of siRNA from endosomes and the ability may depend on different factors, even pH. Different nanoparticles are under study including lipid-based nanoparticles (LNPs), polymeric nanoparticles, carbon nanotubes, gold nanoparticles, hybrid nanoparticles comprised of two cationic polymers as well as cholesterol to self-assemble siRNA into a cationic nanocomplex [144]. The clinical application of RNAi therapy remains limited as siRNA therapeutics must overcome physiological and cellular barriers, hindering siRNA access to the cytoplasm of target cells. Nanoparticles can serve as carriers of siRNA, miRNA, or shRNA through covalent bonds with NP components or through electrostatic interactions with the NP surface, due to their strong negative charge [204].

Metal-based nanoparticles like gold, silver, or iron oxide and carbon materials may be used to deliver siRNA. Hybrid nanoparticles improve the gene delivery efficiency by overcoming the limitations associated with individual methods including transfection efficiency and bioavailability. Iron oxide nanoparticles can be used for the delivery of therapeutic siRNA for noninvasive cancer imaging and treatment. Using exosomes may increase the efficiency of siRNA delivery [197]. Short hairpin RNA (shRNA) may be delivered using for example folate-targeted nanoparticle enhancing radiosensitivity or transferrin-conjugated polyethylene glycol [197].

T cell chimeric antigen receptor (CART) immunotherapy uses polymer nanoparticles to transport DNA—leukemia-targeting CAR genes into the nucleus of the T-cells, thereby providing lasting disease treatment. Besides, nanoparticles prevent the suppression of tumor-infiltrating lymphocytes through the production of adenosine [198].

Naked DNA can be used for the treatment of skin tumors using high-pressure gene guns or through inhalation for lung cancers [195,208]. Another strategy at the nanoscale for cancer treatment may be the use of microRNAs (miRNAs) representing a class of small non-coding and functional RNAs (18–22 nucleotides long) involved in the regulation of many cancer-related genes. These genes have been reported to affect several cell-signaling pathways, which is essential to tumor growth, development, and progression [195,209]. Delivery of miRNAs and anti-miRNAs (anti-miRs) for therapeutic purposes may be connected with conjugation of an RNA nanoparticle with epidermal growth factor receptor (EGFR) aptamer or using ultrasound-induced microbubble cavitation or using exosomes [197]. Another emerging therapy is the use of messenger RNA (mRNA) for transient protein expression and regulation of gene expiration by alternative splicing, through the introduction of a premature stop codon leading to degradation [195].

An interesting strategy for cancer treatment may be inhibition of telomerase activity using different chemical compounds including mechanisms of Pterostilbene-induced senescence through the inhibition of telomerase in lung cancer cells and the perylene diimide derivatives degradation [210,211]. Nucleic acid-based therapy is considered a valuable strategy for the treatment of diseases [208]. Suicide gene therapy is using the delivery of transduced messenger RNA of suicide gene by retrovirus infection released exosomes to deliver the suicide gene to targeted cancer cells and selectively induce apoptosis in tumor cells. Other studies showed that using combined antitumor affecting different genes of cancer cells significantly improve the therapeutic effect than using single-suicide gene therapy [212,213]. Double-suicide gene therapy may induce post-apoptotic necrotic cell death [214].

Exosomes are nanosized lipid bilayer vesicles (30–100 nm) whose small size, cellular origin, flexibility to incorporate macromolecules such as DNA, RNA, and micro-RNA (miRNA) into their lumen, and the ability to cross severe barriers such as the blood-brain barrier enables efficient gene delivery [197].

Nanocarriers may be used as technological innovation for tumor targeting of gene therapeutics [215,216]. Among the most commonly used nanoparticles as carriers should be mentioned: inorganic nanoparticles like gold nanoparticles or mesoporous silica nanoparticles, polymeric nanoparticles including nanocapsules, nanospheres, micelles, nanogels, or dendrimers, and lipid-based nanoparticles with liposomes, solid lipid nanoparticles, and phospholipid micelles [215].

Gold nanoparticles can easily enter cells and have the ability to deliver drugs, genes, and imaging agents with low solubility and poor pharmacokinetics. They come in a variety of shapes and structures such as spheres, rods, stars, and clusters, and are functionalized at the surface with ligands to achieve increased selectivity in tumors and specifically deliver their charge. modification with polymers or linkers containing PEG allows conjugation of complex formation with a drug or siRNA/DNA. They are characterized by high capacity, low toxicity, and efficiency of cell absorption, rapid escape from endosomes, and are stable in the bloodstream [197]. 

Liposome-based nanoparticle systems have been widely used for drug and gene delivery, beginning with the approval in 1995 of the US Food and Drug Administration (FDA) of a liposomal formulation for the chemotherapeutic anticancer doxorubicin, Doxorubicinil^®^ [197]. Due to its lipophilic properties and easy intercalation, Doxorubicin can be internalized by cells by passive diffusion and accumulates intracellularly in high concentrations. Poly(d,l-lactic-co-glycolic) acid (PLGA) and poly(lactic acid) (PLA) may be mentioned among FDA-approved nanopolymers for delivery therapeutic biomolecules [215].

Nanoparticles and nanoplexes used for site-specific delivery of genetic material usually are based on natural, semisynthetic, or synthetic polymers with biocompatible and biodegradable properties. Nanostructured lipid carriers (NLC) enabling the delivery of therapeutics are based on solid and liquid lipids as the core matrix. Provides increased solubility and storage stability, improved permeability and physiological bioavailability, reduced side effects, extended shelf life, and tissue targeted delivery [217,218]. Moreover, the nanoparticle can induce apoptosis in vitro and in vivo and can reduce cardiotoxicity and toxicity to blood cells [218]. Widely used phosphatidylcholine liposomes are non-toxic but are quickly removed by the reticular endothelial system [202,215], they can enhance the immune response to DNA vaccines by increasing their uptake by antigen-presenting cells APCs in lymphoid tissues.

Nanoparticles require critical control of the formulation and process parameters for obtaining desired particle size, zeta potential, entrapment efficiency, and drug release characteristics. An important feature is lack of toxicity and immunogenicity and on the other hand transfection efficiency for internalization into the cells.

Due to their biocompatibility, high moisture content, and desirable mechanical properties, nanogels have unique applications in polymeric carrier systems for bioactive compounds such as DNA, proteins, carbohydrates, and drugs in a polymer lattice, along with their in vitro release pattern.

Using tumor necrosis factor-related apoptosis-inducing ligand (TRAIL or APO2L) in form of TRAIL gene therapy enables specific delivery. Also, combination with other anticancer agents using co-delivery may solve the problem of TRAIL resistance. Liposome-bound TRAIL induces more efficient apoptosis than the soluble form [208]. 

DNA nanococoons are prepared from self-assembling single-stranded DNA building capsules surrounding doxorubicin and deoxyribonuclease (DNase) and are functionalized by folic acid and hyaluronic ligands. DNA nanococoons use a DNA molecular marker for a small delivery system that uses fewer drugs and therefore reduces the side effect [219]. Commercially available bioactive nanocapsules have a high loading capacity, longer dose retention depending on the site, efficient absorption of active substances, increased bioavailability, greater safety and efficacy, and an extended half-life [218].

Carbon nanomaterials (CNMs), including graphene, fullerenes, carbon nanotubes, and carbon quantum dots, load the drug of interest through hydrophobic interactions or π–π stacking to be used as an effective drug genes or proteins delivery platform in cancer therapy [218]. Functionalized carbon nanotubes are widely used as drug carriers for the delivery of small interfering (siRNA), paclitaxel, doxorubicin, flavonoids, among others [16,106,220]. 

Dendrimers are highly branched and have easily modifiable surfaces, they may be conjugated with drugs and nucleic acids (DNA or RNA). Dendrimers with the ability to increase the bioavailability of hydrophobic drugs have added the benefit of trapping drugs with various functional groups. Dendrimers can be used as drug and gene carriers in functional improvisation for drug delivery and anti-cancer therapy, among them polyamidoamine dendrimers (PAMAMs) are the most studied drug delivery [218]. Drugs can be incorporated into cavities or attached to the periphery by chemical means, which enables controlled and defined drug delivery. PAMAM dendrimers can also transfer nucleic acids, aided by the formation of complexes based on electrostatic interactions [197].

For vaccines delivery, nanoemulsions are also used as an example of colloidal dispersions (size 20–200 nm) and the addition of CpG improves the efficiency of vaccines in tumors. Magnetic nanoparticles additionally influence tumor cells by their ability for induction of hyperthermia [221]. Iron oxide nanoparticles, also known as magnetic nanoparticles, enable the attachment or entrapment of a drug or DNA charge in the nanoparticle. Under the influence of an external magnetic field, magnetic nanoparticles are attracted to the tumor and deliver drugs, which can prevent drug build-up in healthy tissue. Superparamagnetic nanoparticles only behave like magnets when this external magnetic field is applied and do not cause toxicity themselves [197]. EDV™ minicells of bacterial origin are used as a nanoplatform for drug/gene encapsulation with specific targeting capability. Bacterial minicells can be used as vectors for chemotherapeutic agents of various charges, structures, solubility, and hydrophobicity.

Encapsulation is by unilateral concentration-dependent diffusion and incubation time with the drug, and specificity is achieved by bispecific antibodies. Minicells are internalized and degraded by endosomes/lysosomes and release the cargo into the cytosol [217].

Other types of carriers are bilayer vesicles called niosomes, containing nonionic surfactants. Niosomes show greater stability, solubility, and bioavailability of bioactive compounds than liposomes and may offer an excellent, inexpensive alternative for drug delivery or gene transfer purposes [222]. Alternative vesicles are bilosomes, nano-vesicular bilamellar carriers composed of bile salt, and nonionic surfactant vesicles for biomedical and pharmaceutical applications [223,224].

Improved immunological properties show archeosomes as liposomes composed of polar phospholipids extracted from archaea, such as methanogens, halophiles, and thermoacidophils. Archeosomes have better stability, are more resistant to chemical hydrolysis, oxidation, bile salts, acidic or basic pH, and high temperatures in a variety of vaccine and drug applications [202]. Archaeosomes are effective for the delivery of antigens through the oral route of delivery to elicit their systemic immune response [215]. The human papillomavirus type 16 genes, containing truncated L1, E6, and E7, were simultaneously used in combination therapy with archaeosome [202]. Archaeosomes promoted immune responses to DNA vaccines and a long-term CTL response was generated with a low antigen dose and induced prophylactic and therapeutic effect against the development of tumor in the animal model [202]. Aptamers as single-stranded RNA or DNA oligonucleotides that selectively bind to cells, tissues, nucleic acids, proteins as well as small molecules have increased target specificity, systemic stability, binding capacity, and low toxicity. Aptamers can bind to one chemical or multiple therapeutic agents, including chemotherapeutic agents and gene silencing agents [225].

Other types of nanocarriers used for gene delivery and cancer vaccines are microspheres as spherical particles with a size of less than 125 nm in diameter [215]. Microspheres are built from polymers derived from the natural, semi-synthetic and synthetic polymers and show great versatility, ranging from small to large, from solid to hollow porous. Microspheres prepared from natural polymers like chitosan, alginate, dextran, among others, primarily exhibit swelling and surface erosion phenomena, while synthetic polymers like PLA and PLGA exhibit bulk corrosion as the principal mechanism for releasing antigen [215]. Microsphere carriers can facilitate the transport of molecules to target cells or tissues, and then release them in a controlled manner [216].

Viral nanovectors usually use nanoparticles from the envelope of viruses due to properties of viruses for internalization in cells, high specificity, overcoming of the biological barriers, longer survival in vivo, and elucidation of the immune systems more efficiently than conventional nanoparticles. Typical viral vectors used in gene delivery include adeno-associated virus, lentivirus, adenovirus, retrovirus, or herpes simplex virus [195]. Viruses are artificially modified to become oncolytic viruses as an effective and potent therapeutic agent for cancer [195,226,227]. Viruses are already used for the treatment of different infections, cancers, and other diseases in the forms of bacteriophages, oncolytic viruses, vector-based viral delivery, virus-like particles, and virosomes. Oncolytic viruses (OV) are used as gene delivery vehicles, enable high transfection efficiency, while nanoparticles provide low immunogenicity and have limited safety concerns. Delivery medium is a critical component of gene therapy [201]. The efficacy of viral therapies has been repeatedly emphasized in clinical trials in virotherapy, gene delivery, and virus-like particles (VLP), with many drugs being approved and marketed [227]. Using viral vectors for nanodelivery has limitations including immunogenicity, toxicity, mutagenicity, and high cost, and limited size of the carrying capacity of some types of viruses [195].

**Figure 7:**
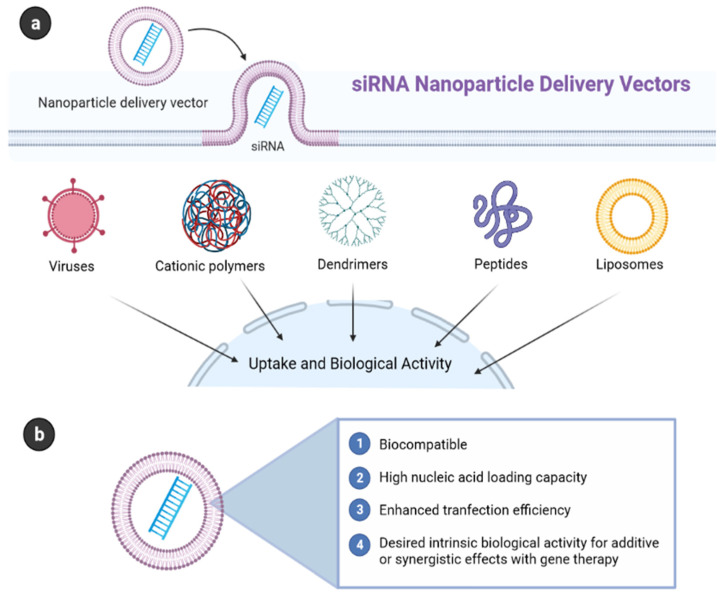
Graphical summary of (**a**) delivery vectors for intracellular delivery of nucleic acids. Apart from viruses, synthetic cationic vectors such as cationic polymers, branched dendrimers, cell-penetrating (CP) peptides, and cationic liposomes can be used to deliver genes into cells; (**b**) Properties of an engineered synthetic vector for gene therapy in the future. In addition to exhibiting good biocompatibility, loading capacity, and transfection efficiency, a future synthetic vector may also be designed to have a desired intrinsic biological activity that would enhance the effects of gene therapy [own drawing].

A schematic representation of delivery vectors for intracellular delivery of nucleic acids, as well as properties of an engineered synthetic vector for gene therapy, were shown in Figure 7.

For the delivery of cancer vaccines, the most frequently used are lipid-based nanocarriers, triglyceride emulsions, solid lipid nanoparticles, and self-emulsifying drug-delivery systems [215]. The antigens or nucleic acids encapsulated within the lipid matrix can easily enter into the lymphatic system via intestinal lymphoid tissues to trigger the immune response through the oral route. Research indicates that lipid nanoemulsions can serve as efficient and impregnated delivery tools with high bioavailability. Nanoemulsions representing a new and promising class of nanocarriers for cancer treatment are equipped with a hydrophobic core that allows encapsulation of lipophilic compounds and efficient uptake by cells of hydrophobic therapeutic agents [218].

Genetic vaccines for cancer treatment are delivered using nanocarriers, which enable high stability, long-lasting, biodegradability, high safety, and strong biocompatibility. The commercial production of cancer vaccines requires optimization of the product and process parameters including antigen loading efficiency, particle size, zeta potential, and controlled release delivery profile of the nanocarriers [215].

The FDA approved a number of nanomedicines-based cancer vaccines based on liposomes, transferases, and microspheres technology, while many others are under the clinical evaluation and translation stage [215]. The therapeutic paradigm of cancer therapy is currently changing from drug delivery to gene delivery.

The detection and isolation of circulating tumor cells (CTCs) in blood or lymph is a non-invasive diagnostic approach to tumor metastasis assessment. Based on nanotechnology, nanocarrier-mediated microfluidic systems, optical aptamer nanoprobes, and NanoFlares have been developed mainly based on overexpression of an exogenous epithelial cell adhesion molecule (EpCAM) or mucin1 to CTC1. Gold nanoparticles may be used [228].

In reducing tumor metastasis, four strategies can be used to prevent tumor metastasis by remodeling the tumor microenvironment; tracking free circulating CTC cancer cells with specific drug delivery strategies; keeping CTCs away from preferred colonization organs, and diagnosis and treatment of metastases. It will be necessary to determine the time required to detect CTCs or remodeling the microenvironment of primary tumors and/or potential metastatic organs. The problem is the heterogeneity of tumors in different patients or the same patient with different disease stages, as well as early diagnosis of metastatic lesions [228].

The use of nanocarriers for the delivery of drugs or genes allows you to avoid problems with solubility and stability of transported charges, prevents degradation, increases the half-life in the systemic circulation, improves the distribution and targeting of action. It enables the long-term release of drugs in the target site, and thanks to the transport of many drugs, it reduces the occurrence of drug resistance [106,220].

Different types of nanoparticles investigated for drug/gene delivery were summarized in Table 2.

## 7. The Impact of Cancer Nanopharmaceuticals on DNA Toxicity

The development of multifunctional nanopharmaceuticals has a huge impact on the use in cancer treatment because they more effectively deliver the drug to specific tissues while reducing the drug’s toxicity. Accordingly, polymeric asparaginase conjugates and polymeric paclitaxel micelles are preferred for the treatment of various types of cancer. Nanotechnology-based therapeutics and diagnostics, including diagnostic imaging, provide greater efficacy with a significant reduction in toxicity. Future solutions will concern the application of nanorobotics in healthcare [218]. Natural polymer nanoparticles are composed of polymers such as alginate, chitosan, albumin, and gelatin. Coating of polymers with polysorbates increases the bioavailability of drugs to the brain by solubilization and fluidization of endothelial cell membranes due to the surfactant effect of polysorbates. Polysorbate nanoparticles increase the bioavailability of drugs into the brain through the blood-brain barrier. Another advantage is increased stability of volatile pharmaceutical agents, improved administration through oral and intravenous methods, and delivery of a higher load of pharmaceutical agents to the desired location. Disadvantages of the polymeric nanoparticles include toxic degradation, toxic monomers aggregation, residual material associated with these nanoparticles, and toxic degradation processes involved in such systems.

The nanocarriers-based strategy has incorporated siRNA molecules with nanoparticulate encapsulation for direct delivery into cells. siRNAs are involved in the regulation of gene expression, thus significantly influencing tumor cells [229].

RNAi is still characterized by short-term bioavailability due to systemic degradation and low tissue permeability. Gene silencing agents can be delivered to target cells using nanocarriers to avoid degradation by serum nucleases, repulsion by cells, problems with low permeability, instability, and low efficiency [225].

Nanoparticles enable the transport of small-molecule compounds, additionally, there is the possibility of functionalization with ligands, including small molecules, DNA or RNA strands, peptides, aptamers, or antibodies. This enables drug delivery, multi-modal therapy, and a combined therapeutic and diagnostic effect termed “theragnostics”. The ability of nanoparticles to absorb energy and re-radiation enables the destruction of diseased tissue through laser ablation and hyperthermia (National Cancer Institute).

Polyethyleneimine (PEI), is commonly used for the delivery of siRNA or DNA for gene therapy as a cationic linear or branched polymer PEI has numerous free cationic groups that can electrostatically interact with nucleic acids and thereby condense them to form nanosized particles. Modification with polyethylene glycol (PEG) reduces the toxicity of PEI to cells and enhances its in vivo stability [197]. Other biocompatible and biodegradable polymers commonly used for gene delivery are cationic polysaccharides chitosan and gelatin. The cationic nature of chitosan facilitates gene delivery since negatively charged nucleic acids easily complex with chitosan to form nanoparticles. Cross-linked gelatin forms stable nanoparticles and increases the delivery efficiency of a variety of drugs, including chemotherapeutic agents, proteins and peptides, and siRNAs [197].

Monoclonal antibodies, fragments thereof, ribonucleic acid/deoxyribonucleic acid aptamers (RNA/DNA aptamers), and peptides are increasingly used. They are believed to increase the sensitivity of therapeutic agents and focus on nanoformulation-based specific delivery [225]. Promoter-bound ribonucleic acids (pRNAs) can be used as anti-cancer therapy. Promoter-bound RNAs (pRNAs) can bind to each other to form a nanocage or ring structure that can be used as a vehicle for the delivery of therapeutics to cancer cells. pRNAs can modulate epigenetic changes in cells that result in the silencing of transcription genes.

Another type of therapy that relies on external electromagnetic irradiation is photodynamic therapy (PDT), which involves locating a photosensitizer tumor and then activating it with light to produce cytotoxic reactive oxygen species (ROS) [230]. Most commonly used are nanomaterials with a high Z-core doped with lanthanide or hafnium, after external exposure to X-rays, the nanoparticle core emits photons of visible light locally at the tumor site. The emission of photons from the particles then activates the nanoparticle-bound or local photosensitizer to generate singlet oxygen (^1^O_2_) ROS for tumor destruction. Nanoparticles can be used for PDT photodynamic therapy, which generates ROS, and for enhanced radiation therapy through a high Z core. A form of Cherenkov radiation can also be used at a similar end of local photon emission to use as a trigger for local PDT. Nanomaterials enhance the stability of DNA and RNA therapeutics such as small interfering RNA (siRNA) and microRNA (miRNA) delivered as capsules or coupled to the surface of nanoparticles in the systemic circulation (National Cancer Institute). An effective RNAi-mediated gene silencing requires overcoming many physiological barriers based on nanoparticles and lipids [204].

The cytotoxic effect of doxorubicin involves the insertion of the flat portion of doxorubicin adriamycinone between adjacent DNA base pairs, which interferes with the enzyme topoisomerase II (TOP2) and stops DNA replication and RNA transcription. Also, it is assumed to produce carbon-concentrated radicals and reactive oxygen species (ROS) that destroy the cell membrane, proteins, and DNA [217].

One popular target of RNA interference is the HER2 gene associated with breast, stomach, ovarian, and colon cancer. RNA interference reduces the expression of the HER2 protein on the cell surface. Switching off the HER2 gene causes the accumulation of cells in the late G1/S phase, leading to growth inhibition and apoptosis. RNA interference is limited in vivo by degradation by ribonuclease [225].

It is assumed that sunscreen preparations based on zinc oxide and titanium dioxide nanoparticles destroy DNA. Intensive work is underway to understand the absorption, distribution, metabolism, and excretion of nanomaterials [218].

shRNA molecules can facilitate long-term silencing of target gene expression by RNAi by providing an intracellular plasmid containing specific shRNA sequences capable of targeting mRNA strands after processing by Dicer. ShRNA plasmids are DNA-based and are more resistant to degradation than dsRNA, however, like miRNAs, they require an expression vector [204,225].

Cationic liposomes are made using positively charged lipids and can interact with negatively charged DNA. They can be used to deliver cancer vaccines by loading synthetically long peptides into liposomes to be delivered to dendritic cells, thus enhancing the immune response [231]. In turn, dendrimers, due to their branched structure, enable specific delivery of drugs and genes at the DNA or RNA level and also ensure specificity of the size and mass of the molecule. Dendrimers can also facilitate the penetration of hydrophilic nucleic acids through the cell membrane [188,189]. 

The size and shape of gold (Au) nanoparticles are believed to influence the transfection efficiency and intracellular distribution of siRNA. The cellular uptake of larger particles with a diameter of 50 nm beads and 40 nm stars was higher compared to the 13 nm beads [106,220].

## 8. Regulatory Aspects

There is much debate about the over-generalization of guidelines and recommendations for the safe handling of nanoscale objects since there are not two nanomaterials with identical properties. Several government agencies and non-governmental organizations have devoted efforts to establish guidelines for innovative testing, characterization, control, and risk assessment of nanomaterials, some of which apply to nanodrugs, nanoadditives, and nanocarriers. The pharmaceutical preparation of a nanodrug has several driving factors; for compounds whose solubility in water or dissolution rate limits their oral bioavailability, reducing the size to the nanoscale can increase the dissolution rate in vivo and the fraction absorbed. Poorly soluble drugs can be ground to a specific size, resulting not only in useful bioavailability but also in the sustained release.

There are two general nanofabrication methods through which the construction of nanomaterials can be envisaged to produce clinically compliant products: the “top-down” and the “bottom-up” approaches [232,233]. The first approach is based on various methods that etch the bulk material (usually via lithography), resulting in materials of smaller size, being highly tunable and uniform. Unfortunately, the smaller nanosystems are anticipated, the bigger associated challenges occur related to the quantum effects. This renders the top-down strategies not as easily controllable as the bottom-up processes, which focus on single atoms or molecules and can be further arranged into sophisticated architectures of the desired size upon harnessing a multitude of covalent and/or supramolecular non-covalent interactions. Through self-assembly and self-organization phenomena, a variety of 2D and 3D functional nanomaterials has been constructed and utilized in domains such as nanoelectronics, sensing, and bioassays [194,234,235].

Another of the current problems in controlling the quality of nanodrugs is the complexity of the characterization of nanoparticles, for example, the particle size can be reported either by the diameter of the aggregate compound or by the size of the primary particle. Thus, databases began to be set/built [236,237,238]. The Nanomaterial Registry (NR) presents a range of properties that define the key characteristics of nanoparticles, which involve not only properties but also measurement techniques and parameters. It is evident that nanoparticles exist in large populations and are found in a variety of environments or conditions, as a result, how data is collected and expressed depends on the following considerations:

How the physical-chemical property is measured;
The way the property is reported;
The measurement technique and the instrument used;
The record in which the sample is collected and prepared for the examination.

Thus, databases can assign confidence measures to data to assist those who use them. This approach is the first step to ensure that there are quality and reliability in the measurement and the data generated. For example, the simple indication of size, without regard to the method, instrument, and procedures used would make it difficult or almost impossible to repeat or compare studies, but to experiment with a known protocol (such as those promulgated by ASTM International or the Nanotechnology Characterization Laboratory within the National Cancer Institute) in which the specifications are provided would be the most reliable and, therefore, would bring a greater degree of confidence to the acquired data. In summary, records can be used to explore public data available to support decision-making for the application of the researcher’s interest. Thus, unnecessary repetition of efforts is minimized in areas where data are numerous and the value of new experimental data obtained is maximized. 

**Figure 8:**
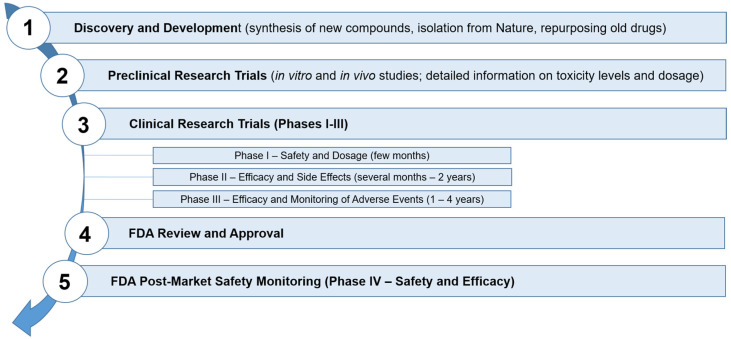
A flowchart representing the drug development process [own drawing].

A flowchart of a drug development process was added to show the process a drug needs to undergo to be approved by the FDA (Figure 8).

Although most of the currently synthesized pharmaceuticals are new systems, it is important to note that the drug discovery process (1) can also benefit from repurposing of well-known drugs or isolation of active substances from Nature. The compound of interest is then subjected to the preclinical research trials (2), which provide detailed information on the needed dosage and related toxicity levels in in vitro and in vivo studies. Successful candidates are subjected to three-phase clinical research trials, which last up to four years, where safety, efficacy, side effects, and adverse events associated with the drug are evaluated. Once the drug is safe and effective, it can undergo the FDA review and eventual approval (4), which commences the Post-Market Safety Monitoring (phase IV). 

Also, comprehensive databases allow gaps to be identified and guide the exploration of compositions, properties, and results of less studied nanomaterials to serve the application of interest, for example, effectiveness, safety, environmental disposition, or risk assessment, expanding thus the general knowledge of nanomaterials for the benefit of the scientific community and society.

## 9. Conclusions

Analytical considerations of nanopharmaceuticals for cancer therapy play an instrumental role in the quality control of a formulation, as therapeutic agents are complex in terms of components, function, and action. The six components that dictate the function and action of nanodrugs are the presence and concentration of the active principle, the surface properties, chemical composition of the drug, physical formulation (solid or liquid), and form of administration. Therefore, all of these parameters are targets for fine-tuning, which ideally should lead to non-toxic, selective, and efficient nanopharmaceuticals. The stabilization of nanopharmaceuticals is another crucial issue, which influences their therapeutic effectiveness. Nanopharmaceuticals have found a range of applications in cancer therapy, including in immunotherapy and gene therapy, for site-specific targeting to reduce systemic toxicity, for controlled release strategies, and even for theragnostic. The potential use of one single device for diagnosis and therapy opens perspectives to overcome multi-drug resistance, shorten the chemotherapeutic regimens, and ultimately improve the quality of patients and caretakers. To achieve this objective, appropriate analytical tools covering the sensitive physicochemical parameters of nanopharmaceuticals need to be critically selected to provide synergistic information for their full in vitro/in vivo characterization. 

## Figures and Tables

**Table 1:** Available clinical trials including usage of gene therapy in cancers as of date 3 April 2021 [ClinicalTrials.gov].

NCT Number	Title	Status	Interventions	Phases
NCT00009841	Gene Therapy in Treating Patients With Advanced Head and Neck Cancer	Completed	Biological: EGFR antisense DNA Biological: growth factor antagonist therapy Drug: DC-cholesterol liposome	Phase 1
NCT00006033	Interleukin-2 Gene or Methotrexate in Treating Patients With Recurrent or Refractory Stage III or Stage IV Head and Neck Cancer	Completed	Biological: gene therapy Biological: interleukin-2 gene Drug: methotrexate	Phase 2
NCT00059605	Phase I Study of IV DOTAP: Cholesterol-Fus1 in Non-Small-Cell Lung Cancer	Completed	Genetic: DOTAP:Chol-fus1	Phase 1
NCT00044993	Chemotherapy Combined With Gene Therapy in Treating Patients Who Have Stage III or Stage IV Breast Cancer	Completed	Biological: Ad5CMV-p53 gene Drug: docetaxel Drug: doxorubicin hydrochloride Procedure: conventional surgery Procedure: neoadjuvant therapy	Phase 2
NCT04486833	TUSC2-nanoparticles (GPX-001) and Osimertinib in Patients With Stage IV Lung Cancer Who Progressed on Osimertinib Alone	Not yet recruiting	Biological: Quaratusugene ozeplasmid—intravenous infusion Drug: Osimertinib Oral Tablet	Phase 1|Phase 2
NCT02337985	Gene Therapy and Combination Chemotherapy in Treating Patients With AIDS-Related Non-Hodgkin Lymphoma	Active, not recruiting	Drug: Prednisone Biological: Rituximab Drug: Etoposide Drug: Doxorubicin Hydrochloride Drug: Vincristine Sulfate Drug: Cyclophosphamide Biological: Filgrastim Biological: Lentivirus Vector rHIV7-shI-TAR-CCR5RZ-transduced Hematopoietic Stem/Progenitor Cells	Phase 1
NCT01591356	EphA2 siRNA in Treating Patients With Advanced or Recurrent Solid Tumors	Recruiting	Drug: EphA2-targeting DOPC-encapsulated siRNA Other: Laboratory Biomarker Analysis Other: Pharmacological Study	Phase 1
NCT02528682	MiHA-loaded PD-L-silenced DC Vaccination After Allogeneic SCT	Completed	Biological: MiHA-loaded PD-L-silenced DC Vaccination	Phase 1|Phase 2
NCT03087591	APN401 in Treating Patients With Recurrent or Metastatic Pancreatic Cancer, Colorectal Cancer, or Other Solid Tumors That Cannot Be Removed by Surgery	Active, not recruiting	Other: Laboratory Biomarker Analysis Biological: siRNA-transfected Peripheral Blood Mononuclear Cells APN401	Phase 1
NCT03608631	iExosomes in Treating Participants With Metastatic Pancreas Cancer With KrasG12D Mutation	Recruiting	Drug: Mesenchymal Stromal Cells-derived Exosomes with KRAS G12D siRNA	Phase 1
NCT04278326	Primary Organoid Models and Combined Nucleic Acids Therapeutics for Anti-HPV Treatments	Recruiting	Procedure: Vaginal Biopsy	Not Applicable

**Table 2:** Different types of nanoparticles investigated for drug/gene delivery.

Particle Class	Materials	Application
Natural materials or derivatives	Liposomes Chitosan Gelatine Dextrane Starch Alginates Metal-based nanoparticles	Drug/gene delivery
Dendrimers	Branched polymers	Drug delivery/gene delivery
Polymer carriers	Block copolymers Polylactic acid Polycaprolactone Polyethyleneimine Poly(cyano)acrylates	Drug/gene delivery
Nucleic acids	Micro RNA (miRNA) Small interfering RNA (siRNA) Oligonucleotides CRISPR/Cas9 Short hairpin RNA (shRNA)	Gene therapy RNA interference therapy
Non-viral vectors	Physically mediated methods (microinjection, ultrasound-mediated microbubble, microparticle bombardment, and electroporation) Chemical vectors (cationic polymers and cationic liposomes, shell nanoparticles, and polymeric nanoparticles), Biological methods (bacteria and specific mammalian cells).	Gene delivery
Viral vectors	Adenovirus-associated virus Lentivirus	cell-based gene therapy
Vesicles	Exosomes Bilosomes Niosomes Archeosomes minicells	Drug/gene delivery
Various	Silica-nanoparticles Mixtures of above	Gene delivery Reversal of tumor acidity

## Data Availability

Not applicable.

## References

[1] Hartshorn C.M., Bradbury M.S., Lanza G.M., Nel A.E., Rao J., Wang A.Z., Wiesner U.B., Yang L., Grodzinski P. (2018). Nanotechnology Strategies To Advance Outcomes in Clinical Cancer Care. ACS Nano.

[2] Cryer A.M., Thorley A.J. (2019). Nanotechnology in the diagnosis and treatment of lung cancer. Pharmacol. Ther..

[3] Krishnan S.R., George S.K. (2014). Nanotherapeutics in cancer prevention, diagnosis and treatment. Pharmacology and Therapeutics.

[4] Parvanian S., Mostafavi S.M., Aghashiri M. (2017). Multifunctional nanoparticle developments in cancer diagnosis and treatment. Sens. BioSens. Res..

[5] Wang S.-Y., Hu H.-Z., Qing X.-C., Zhang Z.-C., Shao Z.-W. (2020). Recent advances of drug delivery nanocarriers in osteosarcoma treatment. J. Cancer.

[6] Singhvi G., Rapalli V.K., Nagpal S., Dubey S.K., Saha R.N. (2020). Nanocarriers as potential targeted drug delivery for cancer therapy. Nanoscience in Medicine.

[7] Souto E.B., Doktorovova S., Campos J.R., Martins-Lopes P., Silva A.M. (2019). Surface-tailored anti-HER2/neu-solid lipid nanoparticles for site-specific targeting MCF-7 and BT-474 breast cancer cells. Eur. J. Pharm. Sci..

[8] Sanchez-Lopez E., Guerra M., Dias-Ferreira J., Lopez-Machado A., Ettcheto M., Cano A., Espina M., Camins A., Garcia M.L., Souto E.B. (2019). Current Applications of Nanoemulsions in Cancer Therapeutics. Nanomaterials.

[9] Yu D., Zhang N., Liu S., Hu W., Nie J.-J., Zhang K., Yu B., Wang Z.-G., Xu F.-J. (2020). Self-Assembled Nucleotide/Saccharide-Tethering Polycation-Based Nanoparticle for Targeted Tumor Therapy. ACS Mater. Lett..

[10] Wankar J., Kotla N.G., Gera S., Rasala S., Pandit A., Rochev Y.A. (2020). Recent advances in host–guest self-assembled cyclodextrin carriers: Implications for responsive drug delivery and biomedical engineering. Adv. Funct. Mater..

[11] Avramović N., Mandić B., Savić-Radojević A., Simić T. (2020). Polymeric Nanocarriers of Drug Delivery Systems in Cancer Therapy. Pharmaceutics.

[12] Zielińska A., Carreiró F., Oliveira A., Neves A., Pires B., Venkatesh D., Durazzo A., Lucarini M., Eder P., Silva A. (2020). Polymeric Nanoparticles: Production, Characterization, Toxicology and Ecotoxicology. Molecules.

[13] Jose S., Cinu T.A., Sebastian R., Shoja M.H., Aleykutty N.A., Durazzo A., Lucarini M., Santini A., Souto E.B. (2019). Transferrin-Conjugated Docetaxel-PLGA Nanoparticles for Tumor Targeting: Influence on MCF-7 Cell Cycle. Polymers.

[14] Elzoghby A.O., Hemasa A.L., Freag M.S. (2016). Hybrid protein-inorganic nanoparticles: From tumor-targeted drug delivery to cancer imaging. J. Control. Release.

[15] Kundu M., Chatterjee S., Ghosh N., Manna P., Das J., Sil P.C. (2020). Tumor targeted delivery of umbelliferone via a smart mesoporous silica nanoparticles controlled-release drug delivery system for increased anticancer efficiency. Mater. Sci. Eng. C.

[16] Morais R.P., Novais G.B., Sangenito L.S., Santos A.L.S., Priefer R., Morsink M., Mendonça M.C., Souto E.B., Severino P., Cardoso J.C. (2020). Naringenin-Functionalized Multi-Walled Carbon Nanotubes: A Potential Approach for Site-Specific Remote-Controlled Anticancer Delivery for the Treatment of Lung Cancer Cells. Int. J. Mol. Sci..

[17] Kuchur O., Tsymbal S., Shestovskaya M., Serov N., Dukhinova M., Shtil A. (2020). Metal-derived nanoparticles in tumor theranostics: Potential and limitations. J. Inorg. Biochem..

[18] Muller R.H., Runge S., Ravelli V., Mehnert W., Thunemann A.F., Souto E.B. (2006). Oral bioavailability of cyclosporine: Solid lipid nanoparticles (SLN) versus drug nanocrystals. Int. J. Pharm..

[19] Teeranachaideekul V., Junyaprasert V.B., Souto E.B., Muller R.H. (2008). Development of ascorbyl palmitate nanocrystals applying the nanosuspension technology. Int. J. Pharm..

[20] Aisida S.O., Akpa P.A., Ahmad I., Zhao T.-K., Maaza M., Ezema F.I. (2020). Bio-inspired encapsulation and functionalization of iron oxide nanoparticles for biomedical applications. Eur. Polym. J..

[21] Zhi D., Yang T., Yang J., Fu S., Zhang S. (2020). Targeting strategies for superparamagnetic iron oxide nanoparticles in cancer therapy. Acta Biomater..

[22] El-Boubbou K. (2018). Magnetic iron oxide nanoparticles as drug carriers: Clinical relevance. Nanomedicine.

[23] Lin P.-C., Lin S., Wang P.C., Sridhar R. (2014). Techniques for physicochemical characterization of nanomaterials. Biotechnol. Adv..

[24] Arias J.L. (2011). Advanced methodologies to formulate nanotheragnostic agents for combined drug delivery and imaging. Expert Opin. Drug Deliv..

[25] Mourdikoudis S., Pallares R.M., Thanh N.T. (2018). Characterization techniques for nanoparticles: Comparison and complementarity upon studying nanoparticle properties. Nanoscale.

[26] Spiliopoulou M., Valmas A., Triandafillidis D.-P., Kosinas C., Fitch A., Karavassili F., Margiolaki I. (2020). Applications of X-ray powder diffraction in protein crystallography and drug screening. Crystals.

[27] Gallagher J.R., Li T., Zhao H., Liu J., Lei Y., Zhang X., Ren Y., Elam J.W., Meyer R.J., Winans R.E. (2014). In situ diffraction of highly dispersed supported platinum nanoparticles. Catal. Sci. Technol..

[28] Letzel A., Gökce B., Menzel A., Plech A., Barcikowski S. (2018). Primary particle diameter differentiation and bimodality identification by five analytical methods using gold nanoparticle size distributions synthesized by pulsed laser ablation in liquids. Appl. Surf. Sci..

[29] Zielińska A., Ferreira N.R., Feliczak-Guzik A., Nowak I., Souto E.B. (2020). Loading, release profile and accelerated stability assessment of monoterpenes-loaded Solid Lipid Nanoparticles (SLN). Pharm. Dev. Technol..

[30] Li T., Senesi A.J., Lee B. (2016). Small angle X-ray scattering for nanoparticle research. Chem. Rev..

[31] Mao Y., Liu K., Zhan C., Geng L., Chu B., Hsiao B.S. (2017). Characterization of nanocellulose using small-angle neutron, X-ray, and dynamic light scattering techniques. J. Phys. Chem. B.

[32] Hassan P.A., Rana S., Verma G. (2015). Making sense of Brownian motion: Colloid characterization by dynamic light scattering. Langmuir.

[33] Pawłowska S., Kowalewski T., Pierini F. (2018). Fibrous polymer nanomaterials for biomedical applications and their transport by fluids: An overview. Soft Matter.

[34] Kureha T., Minato H., Suzuki D., Urayama K., Shibayama M. (2019). Concentration dependence of the dynamics of microgel suspensions investigated by dynamic light scattering. Soft Matter.

[35] Wyatt P.J. (1993). Light scattering and the absolute characterization of macromolecules. Anal. Chim. Acta.

[36] Sivakumaran M., Platt M. (2016). Tunable resistive pulse sensing: Potential applications in nanomedicine. Nanomedicine.

[37] Temel D.B., Kinderman F., Eryilmaz E., Houde D.J., Berkowitz S.A. (2020). Developability in biophysical characterization. Biophysical Characterization of Proteins in Developing Biopharmaceuticals.

[38] Minton A.P. (2016). Recent applications of light scattering measurement in the biological and biopharmaceutical sciences. Anal. Biochem..

[39] Tofail S.A., Bauer J. (2016). Electrically polarized biomaterials. Adv. Mater..

[40] Zielińska A., Ferreira N.R., Durazzo A., Lucarini M., Cicero N., Mamouni S.E., Silva A.M., Nowak I., Santini A., Souto E.B. (2019). Development and optimization of alpha-pinene-loaded solid lipid nanoparticles (SLN) using experimental factorial design and dispersion analysis. Molecules.

[41] Manaia E.B., Abuçafy M.P., Chiari-Andréo B.G., Silva B.L., Oshiro Junior J.A., Chiavacci L.A. (2017). Physicochemical characterization of drug nanocarriers. Int. J. Nanomed..

[42] Rasmussen M.K., Pedersen J.N., Marie R. (2020). Size and surface charge characterization of nanoparticles with a salt gradient. Nat. Commun..

[43] Corbett J.C., McNeil-Watson F., Jack R.O., Howarth M. (2012). Measuring surface zeta potential using phase analysis light scattering in a simple dip cell arrangement. Colloids Surf. A Physicochem. Eng. Asp..

[44] Xu R. (2008). Progress in nanoparticles characterization: Sizing and zeta potential measurement. Particuology.

[45] Zhu M., Wang H., Keller A.A., Wang T., Li F. (2014). The effect of humic acid on the aggregation of titanium dioxide nanoparticles under different pH and ionic strengths. Sci. Total Environ..

[46] Klang V., Matsko N.B., Valenta C., Hofer F. (2012). Electron microscopy of nanoemulsions: An essential tool for characterisation and stability assessment. Micron.

[47] Nellist M.R., Chen Y., Mark A., Gödrich S., Stelling C., Jiang J., Poddar R., Li C., Kumar R., Papastavrou G. (2017). Atomic force microscopy with nanoelectrode tips for high resolution electrochemical, nanoadhesion and nanoelectrical imaging. Nanotechnology.

[48] Spyratou E., Makropoulou M., Mourelatou E., Demetzos C. (2012). Biophotonic techniques for manipulation and characterization of drug delivery nanosystems in cancer therapy. Cancer Lett..

[49] Korolkov V.V., Summerfield A., Murphy A., Amabilino D.B., Watanabe K., Taniguchi T., Beton P.H. (2019). Ultra-high resolution imaging of thin films and single strands of polythiophene using atomic force microscopy. Nat. Commun..

[50] Bhushan B. (2007). Handbook of Nano-Technology.

[51] Oliveira Brett A.M., Chiorcea A.-M. (2003). Atomic force microscopy of DNA immobilized onto a highly oriented pyrolytic graphite electrode surface. Langmuir.

[52] Binnig G., Rohrer H., Gerber C., Weibel E. (1982). Surface studies by scanning tunneling microscopy. Phys. Rev. Lett..

[53] Wiesendanger R., Roland W. (1994). Scanning Probe Microscopy and Spectroscopy: Methods and Applications.

[54] Biscarini F., Ong Q.K., Albonetti C., Liscio F., Longobardi M., Mali K.S., Ciesielski A., Reguera J., Renner C., De Feyter S. (2013). Quantitative analysis of scanning tunneling microscopy images of mixed-ligand-functionalized nanoparticles. Langmuir.

[55] Ong Q.K., Reguera J., Silva P.J., Moglianetti M., Harkness K., Longobardi M., Mali K.S., Renner C., De Feyter S., Stellacci F. (2013). High-resolution scanning tunneling microscopy characterization of mixed monolayer protected gold nanoparticles. ACS Nano.

[56] Kano S., Tada T., Majima Y. (2015). Nanoparticle characterization based on STM and STS. Chem. Soc. Rev..

[57] Hansma P., Elings V., Marti O., Bracker C. (1988). Scanning tunneling microscopy and atomic force microscopy: Application to biology and technology. Science.

[58] Wu X., Delbianco M., Anggara K., Michnowicz T., Pardo-Vargas A., Bharate P., Sen S., Pristl M., Rauschenbach S., Schlickum U. (2020). Imaging single glycans. Nature.

[59] Erdal M.S., Güngör S. (2020). Electrospun Nanofibers as Carriers in Dermal Drug Delivery. Nanopharmaceuticals: Principles and Applications.

[60] Astruc D. (2020). Introduction: Nanoparticles in catalysis. Chem. Rev..

[61] Mansfield E., Tewary V.K., Zhang Y. (2015). 6—Recent advances in thermal analysis of nanoparticles: Methods, models and kinetics. Modeling, Characterization, and Production of Nanomaterials.

[62] Cavendish M., Nalone L., Barbosa T., Barbosa R., Costa S., Nunes R., da Silva C.F., Chaud M.V., Souto E.B., Hollanda L. (2019). Study of pre-formulation and development of solid lipid nanoparticles containing perillyl alcohol. J. Therm. Anal. Calorim..

[63] Sanchez-Lopez E., Egea M.A., Cano A., Espina M., Calpena A.C., Ettcheto M., Camins A., Souto E.B., Silva A.M., Garcia M.L. (2016). PEGylated PLGA nanospheres optimized by design of experiments for ocular administration of dexibuprofen-in vitro, ex vivo and in vivo characterization. Colloids Surf. B Biointerfaces.

[64] Montenegro L., Castelli F., Sarpietro M.G. (2018). Differential scanning calorimetry analyses of idebenone-loaded solid lipid nanoparticles interactions with a model of bio-membrane: A comparison with in vitro skin permeation data. Pharmaceuticals.

[65] Kunc F., Balhara V., Sun Y., Daroszewska M., Jakubek Z.J., Hill M., Brinkmann A., Johnston L.J. (2019). Quantification of surface functional groups on silica nanoparticles: Comparison of thermogravimetric analysis and quantitative NMR. Analyst.

[66] Biedunkiewicz A., Gabriel U., Figiel P., Grzesiak D. (2010). Application of thermal analysis in nanotechnology. J. Therm. Anal. Calorim..

[67] Singh S.C., Zeng H., Guo C., Cai W. (2012). Nanomaterials: Processing and Characterization with Lasers.

[68] Jazani S., Sgouralis I., Shafraz O.M., Levitus M., Sivasankar S., Pressé S. (2019). An alternative framework for fluorescence correlation spectroscopy. Nat. Commun..

[69] Ding S.-Y., Yi J., Li J.-F., Ren B., Wu D.-Y., Panneerselvam R., Tian Z.-Q. (2016). Nanostructure-based plasmon-enhanced Raman spectroscopy for surface analysis of materials. Nat. Rev. Mater..

[70] Dendisová M., Jeništová A., Parchaňská-Kokaislová A., Matějka P., Prokopec V., Švecová M. (2018). The use of infrared spectroscopic techniques to characterize nanomaterials and nanostructures: A review. Anal. Chim. Acta.

[71] Li Y.-S., Church J.S. (2014). Raman spectroscopy in the analysis of food and pharmaceutical nanomaterials. J. Food Drug Anal..

[72] Kauffmann T.H., Kokanyan N., Fontana M.D. (2019). Use of Stokes and anti-Stokes Raman scattering for new applications. J. Raman Spectrosc..

[73] Geraldes C.F. (2020). Introduction to Infrared and Raman-Based Biomedical Molecular Imaging and Comparison with Other Modalities. Molecules.

[74] Santos D.I., Neiva Correia M.J., Mateus M.M., Saraiva J.A., Vicente A.A., Moldão M. (2019). Fourier transform infrared (FT-IR) spectroscopy as a possible rapid tool to evaluate abiotic stress effects on pineapple by-products. Appl. Sci..

[75] Yao H., Wynendaele E., Xu X., Kosgei A., De Spiegeleer B. (2018). Circular dichroism in functional quality evaluation of medicines. J. Pharm. Biomed. Anal..

[76] Fasman G.D. (2013). Circular Dichroism and the Conformational Analysis of Biomolecules.

[77] Kumar M., Jha A. (2019). Pharmaceutical Applications of Circular Dichroism for Nanomaterial’s. Adv. Clin. Toxicol..

[78] Spaeth P., Adhikari S., Le L., Jollans T., Pud S., Albrecht W., Bauer T., Caldarola M., Kuipers L., Orrit M. (2019). Circular dichroism measurement of single metal nanoparticles using photothermal imaging. Nano Lett..

[79] Bogachev Y.V., Chernenco J.S., Gareev K., Kononova I., Matyushkin L., Moshnikov V., Nalimova S. (2014). The study of aggregation processes in colloidal solutions of magnetite–silica nanoparticles by NMR relaxometry, AFM, and UV–vis-spectroscopy. Appl. Magn. Reson..

[80] Marbella L.E., Millstone J.E. (2015). NMR techniques for noble metal nanoparticles. Chem. Mater..

[81] Estelrich J., Sánchez-Martín M.J., Busquets M.A. (2015). Nanoparticles in magnetic resonance imaging: From simple to dual contrast agents. Int. J. Nanomed..

[82] Shariatgorji M., Svenningsson P., Andrén P.E. (2014). Mass spectrometry imaging, an emerging technology in neuropsychopharmacology. Neuropsychopharmacology.

[83] Siuzdak G. (2004). An introduction to mass spectrometry ionization: An excerpt from the expanding role of mass spectrometry in biotechnology. JALA J. Assoc. Lab. Autom..

[84] Abraham J., Sharika T., Mishra R.K., Thomas S., Mishra R.K., Thomas S., Kalarikkal N. (2017). 14—Rheological characteristics of nanomaterials and nanocomposites. Micro and Nano Fibrillar Composites (MFCs and NFCs) from Polymer Blends.

[85] Zhang J., Li Y., An F.-F., Zhang X., Chen X., Lee C.-S. (2015). Preparation and size control of sub-100 nm pure nanodrugs. Nano Lett..

[86] Patra J.K., Das G., Fraceto L.F., Campos E.V.R., Rodriguez-Torres M.D.P., Acosta-Torres L.S., Diaz-Torres L.A., Grillo R., Swamy M.K., Sharma S. (2018). Nano based drug delivery systems: Recent developments and future prospects. J. Nanobiotechnol..

[87] De Jong W.H., Borm P.J.A. (2008). Drug delivery and nanoparticles:applications and hazards. Int. J. Nanomed..

[88] Jokerst J.V., Lobovkina T., Zare R.N., Gambhir S.S. (2011). Nanoparticle PEGylation for imaging and therapy. Nanomedicine.

[89] Suk J.S., Xu Q., Kim N., Hanes J., Ensign L.M. (2016). PEGylation as a strategy for improving nanoparticle-based drug and gene delivery. Adv. Drug Deliv. Rev..

[90] Hoang Thi T.T., Pilkington E.H., Nguyen D.H., Lee J.S., Park K.D., Truong N.P. (2020). The importance of poly (ethylene glycol) alternatives for overcoming PEG immunogenicity in drug delivery and bioconjugation. Polymers.

[91] Knop K., Hoogenboom R., Fischer D., Schubert U.S. (2010). Poly (ethylene glycol) in drug delivery: Pros and cons as well as potential alternatives. Angew. Chem. Int. Ed..

[92] Depp V., Alikhani A., Grammer V., Lele B.S. (2009). Native protein-initiated ATRP: A viable and potentially superior alternative to PEGylation for stabilizing biologics. Acta Biomater..

[93] Bazak R., Houri M., Achy S.E., Hussein W., Refaat T. (2014). Passive targeting of nanoparticles to cancer: A comprehensive review of the literature. Mol. Clin. Oncol..

[94] Rosenblum D., Joshi N., Tao W., Karp J.M., Peer D. (2018). Progress and challenges towards targeted delivery of cancer therapeutics. Nat. Commun..

[95] Sanna V., Pala N., Sechi M. (2014). Targeted therapy using nanotechnology: Focus on cancer. Int. J. Nanomed..

[96] Cano A., Ettcheto M., Chang J.H., Barroso E., Espina M., Kuhne B.A., Barenys M., Auladell C., Folch J., Souto E.B. (2019). Dual-drug loaded nanoparticles of Epigallocatechin-3-gallate (EGCG)/Ascorbic acid enhance therapeutic efficacy of EGCG in a APPswe/PS1dE9 Alzheimer’s disease mice model. J Control Release.

[97] Bamrungsap S., Zhao Z., Chen T., Wang L., Li C., Fu T., Tan W. (2012). Nanotechnology in therapeutics: A focus on nanoparticles as a drug delivery system. Nanomedicine.

[98] Yetisgin A.A., Cetinel S., Zuvin M., Kosar A., Kutlu O. (2020). Therapeutic nanoparticles and their targeted delivery applications. Molecules.

[99] Zielińska A., Costa B., Ferreira M.V., Miguéis D., Louros J., Durazzo A., Lucarini M., Eder P., Chaud M.V., Morsink M. (2020). Nanotoxicology and nanosafety: Safety-by-design and testing at a glance. Int. J. Environ. Res. Public Health.

[100] Koury J., Lucero M., Cato C., Chang L., Geiger J., Henry D., Hernandez J., Hung F., Kaur P., Teskey G. (2018). Immunotherapies: Exploiting the Immune System for Cancer Treatment. J. Immunol. Res..

[101] Melero I., Gaudernack G., Gerritsen W., Huber C., Parmiani G., Scholl S., Thatcher N., Wagstaff J., Zielinski C., Faulkner I. (2014). Therapeutic vaccines for cancer: An overview of clinical trials. Nat. Rev. Clin. Oncol..

[102] Jeanbart L., Swartz M.A. (2015). Engineering opportunities in cancer immunotherapy. Proc. Natl. Acad. Sci. USA.

[103] Chen D.S., Mellman I. (2013). Oncology meets immunology: The cancer-immunity cycle. Immunity.

[104] Dong D., Zheng L., Lin J., Zhang B., Zhu Y., Li N., Xie S., Wang Y., Gao N., Huang Z. (2019). Structural basis of assembly of the human T cell receptor–CD3 complex. Nature.

[105] Yarchoan M., Johnson B.A., Lutz E.R., Laheru D.A., Jaffee E.M. (2017). Targeting neoantigens to augment antitumour immunity. Nat. Rev. Cancer.

[106] Navya P.N., Kaphle A., Srinivas S.P., Bhargava S.K., Rotello V.M., Daima H.K. (2019). Current trends and challenges in cancer management and therapy using designer nanomaterials. Nano Converg..

[107] Zhao C.-Y., Cheng R., Yang Z., Tian Z.-M. (2018). Nanotechnology for Cancer Therapy Based on Chemotherapy. Molecules.

[108] Srinivasarao M., Low P.S. (2017). Ligand-targeted drug delivery. Chem. Rev..

[109] Large D.E., Soucy J.R., Hebert J., Auguste D.T. (2019). Advances in receptor-mediated, tumor-targeted drug delivery. Adv. Ther..

[110] Fu X., Shi Y., Qi T., Qiu S., Huang Y., Zhao X., Sun Q., Lin G. (2020). Precise design strategies of nanomedicine for improving cancer therapeutic efficacy using subcellular targeting. Signal Transduct. Target. Ther..

[111] Lu J., Jiang F., Lu A., Zhang G. (2016). Linkers Having a Crucial Role in Antibody-Drug Conjugates. Int. J. Mol. Sci..

[112] Martin T.A., Ye L., Sanders A.J., Lane J., Jiang W.G. (2013). Cancer invasion and metastasis: Molecular and cellular perspective. Madame Curie Bioscience Database [Internet].

[113] Mitchell M.J., Billingsley M.M., Haley R.M., Wechsler M.E., Peppas N.A., Langer R. (2020). Engineering precision nanoparticles for drug delivery. Nat. Rev. Drug Discov..

[114] Maeda H., Nakamura H., Fang J. (2013). The EPR effect for macromolecular drug delivery to solid tumors: Improvement of tumor uptake, lowering of systemic toxicity, and distinct tumor imaging in vivo. Adv. Drug Deliv. Rev..

[115] Yu B., Tai H.C., Xue W., Lee L.J., Lee R.J. (2010). Receptor-targeted nanocarriers for therapeutic delivery to cancer. Mol. Membr. Biol..

[116] Cho K., Wang X., Nie S., Shin D.M. (2008). Therapeutic nanoparticles for drug delivery in cancer. Clin. Cancer Res..

[117] Friedman A.D., Claypool S.E., Liu R. (2013). The smart targeting of nanoparticles. Curr. Pharm. Des..

[118] Yau T., Dan X., Ng C.C.W., Ng T.B. (2015). Lectins with potential for anti-cancer therapy. Molecules.

[119] Verma M., Shukla A.K., Acharya A. (2020). Lectin Nanoconjugates for Targeted Therapeutic Applications. Nanomaterial-Based Biomedical Applications in Molecular Imaging, Diagnostics and Therapy.

[120] David A., Kopecková P., Kopecek J., Rubinstein A. (2002). The role of galactose, lactose, and galactose valency in the biorecognition of N-(2-hydroxypropyl) methacrylamide copolymers by human colon adenocarcinoma cells. Pharm. Res..

[121] Beyer V.P., Monaco A., Napier R., Yilmaz G., Becer C.R. (2020). Bottlebrush Glycopolymers from 2-Oxazolines and Acrylamides for Targeting Dendritic Cell-Specific Intercellular Adhesion Molecule-3-Grabbing Nonintegrin and Mannose-Binding Lectin. Biomacromolecules.

[122] Frigerio B., Bizzoni C., Jansen G., Leamon C.P., Peters G.J., Low P.S., Matherly L.H., Figini M. (2019). Folate Receptors and Transporters: Biological Role and Diagnostic/Therapeutic Targets in Cancer and Other Diseases.

[123] Ledermann J., Canevari S., Thigpen T. (2015). Targeting the folate receptor: Diagnostic and therapeutic approaches to personalize cancer treatments. Ann. Oncol..

[124] Zwicke G.L., Ali Mansoori G., Jeffery C.J. (2012). Utilizing the folate receptor for active targeting of cancer nanotherapeutics. Nano Rev..

[125] Fernández M., Javaid F., Chudasama V. (2018). Advances in targeting the folate receptor in the treatment/imaging of cancers. Chem. Sci..

[126] Bahrami B., Mohammadnia-Afrouzi M., Bakhshaei P., Yazdani Y., Ghalamfarsa G., Yousefi M., Sadreddini S., Jadidi-Niaragh F., Hojjat-Farsangi M. (2015). Folate-conjugated nanoparticles as a potent therapeutic approach in targeted cancer therapy. Tumor Biol..

[127] Kato T., Jin C.S., Ujiie H., Lee D., Fujino K., Wada H., Hu H.-p., Weersink R.A., Chen J., Kaji M. (2017). Nanoparticle targeted folate receptor 1-enhanced photodynamic therapy for lung cancer. Lung Cancer.

[128] Morales-Cruz M., Delgado Y., Castillo B., Figueroa C.M., Molina A.M., Torres A., Milián M., Griebenow K. (2019). Smart targeting to improve cancer therapeutics. Drug Des. Dev. Ther..

[129] Acharya S., Dilnawaz F., Sahoo S.K. (2009). Targeted epidermal growth factor receptor nanoparticle bioconjugates for breast cancer therapy. Biomaterials.

[130] Arslan F.B., Atar K.O., Calis S. (2021). Antibody-mediated drug delivery. Int. J. Pharm..

[131] Arbuthnot P. (2015). Antiviral Gene Therapy: Summary and Perspectives. Gene Ther. Viral Infect..

[132] Zhou J., Rossi J. (2017). Aptamers as targeted therapeutics: Current potential and challenges. Nat. Rev. Drug Discov..

[133] Doktorovova S., Shegokar R., Rakovsky E., Gonzalez-Mira E., Lopes C.M., Silva A.M., Martins-Lopes P., Muller R.H., Souto E.B. (2011). Cationic solid lipid nanoparticles (cSLN): Structure, stability and DNA binding capacity correlation studies. Int. J. Pharm..

[134] Pangburn T.O., Petersen M.A., Waybrant B., Adil M.M., Kokkoli E. (2009). Peptide-and aptamer-functionalized nanovectors for targeted delivery of therapeutics. J. Biomech. Eng..

[135] Zhou J., Rao L., Yu G., Cook T.R., Chen X., Huang F. (2021). Supramolecular cancer nanotheranostics. Chem. Soc. Rev..

[136] Yu C., Hu Y., Duan J., Yuan W., Wang C., Xu H., Yang X.-D. (2011). Novel aptamer-nanoparticle bioconjugates enhances delivery of anticancer drug to MUC1-positive cancer cells in vitro. PLoS ONE.

[137] Drolet D.W., Green L.S., Gold L., Janjic N. (2016). Fit for the Eye: Aptamers in Ocular Disorders. Nucleic Acid Ther..

[138] Ni S., Zhuo Z., Pan Y., Yu Y., Li F., Liu J., Wang L., Wu X., Li D., Wan Y. (2021). Recent Progress in Aptamer Discoveries and Modifications for Therapeutic Applications. ACS Appl. Mater. Interfaces.

[139] He X.W., Liu T., Chen Y.X., Cheng D.J., Li X.R., Xiao Y., Feng Y.L. (2008). Calcium carbonate nanoparticle delivering vascular endothelial growth factor-C siRNA effectively inhibits lymphangiogenesis and growth of gastric cancer in vivo. Cancer Gene Ther..

[140] He X.W., Liu T., Xiao Y., Feng Y.L., Cheng D.J., Tingting G., Zhang L., Zhang Y., Chen Y.X., Tingting G. (2009). Vascular endothelial growth factor-C siRNA delivered via calcium carbonate nanoparticle effectively inhibits lymphangiogenesis and growth of colorectal cancer in vivo. Cancer Biother. Radiopharm..

[141] Luo X., Peng X., Hou J., Wu S., Shen J., Wang L. (2017). Folic acid-functionalized polyethylenimine superparamagnetic iron oxide nanoparticles as theranostic agents for magnetic resonance imaging and PD-L1 siRNA delivery for gastric cancer. Int. J. Nanomed..

[142] Wang Y., Xu Z., Guo S., Zhang L., Sharma A., Robertson G.P., Huang L. (2013). Intravenous delivery of siRNA targeting CD47 effectively inhibits melanoma tumor growth and lung metastasis. Mol. Ther..

[143] Teleanu R.I., Chircov C., Grumezescu A.M., Teleanu D.M. (2019). Tumor Angiogenesis and Anti-Angiogenic Strategies for Cancer Treatment. J. Clin. Med..

[144] Saw P.E., Song E.-W. (2019). Phage display screening of therapeutic peptide for cancer targeting and therapy. Protein Cell.

[145] Greish K. (2010). Enhanced permeability and retention (EPR) effect for anticancer nanomedicine drug targeting. Methods Mol. Biol..

[146] Mitra A.K., Agrahari V., Mandal A., Cholkar K., Natarajan C., Shah S., Joseph M., Trinh H.M., Vaishya R., Yang X. (2015). Novel delivery approaches for cancer therapeutics. J. Control. Release Off. J. Control. Release Soc..

[147] Sanchez-Lopez E., Ettcheto M., Egea M.A., Espina M., Cano A., Calpena A.C., Camins A., Carmona N., Silva A.M., Souto E.B. (2018). Memantine loaded PLGA PEGylated nanoparticles for Alzheimer’s disease: In vitro and in vivo characterization. J Nanobiotechnology.

[148] Tripathi P.P., Arami H., Banga I., Gupta J., Gandhi S. (2018). Cell penetrating peptides in preclinical and clinical cancer diagnosis and therapy. Oncotarget.

[149] Dufès C., Al Robaian M., Somani S. (2013). Transferrin and the transferrin receptor for the targeted delivery of therapeutic agents to the brain and cancer cells. Ther. Deliv..

[150] Li H., Qian Z.M. (2002). Transferrin/transferrin receptor-mediated drug delivery. Med. Res. Rev..

[151] Clark A.J., Davis M.E. (2015). Increased brain uptake of targeted nanoparticles by adding an acid-cleavable linkage between transferrin and the nanoparticle core. Proc. Natl. Acad. Sci. USA.

[152] Goswami U., Dutta A., Raza A., Kandimalla R., Kalita S., Ghosh S.S., Chattopadhyay A. (2018). Transferrin–copper nanocluster–doxorubicin nanoparticles as targeted theranostic cancer Nanodrug. ACS Appl. Mater. Interfaces.

[153] Soe Z.C., Kwon J.B., Thapa R.K., Ou W., Nguyen H.T., Gautam M., Oh K.T., Choi H.-G., Ku S.K., Yong C.S. (2019). Transferrin-conjugated polymeric nanoparticle for receptor-mediated delivery of doxorubicin in doxorubicin-resistant breast cancer cells. Pharmaceutics.

[154] Baskar R., Yap S.P., Chua K.L., Itahana K. (2012). The diverse and complex roles of radiation on cancer treatment: Therapeutic target and genome maintenance. Am. J. Cancer Res..

[155] Pei D., Buyanova M. (2019). Overcoming Endosomal Entrapment in Drug Delivery. Bioconjug. Chem..

[156] Bonam S.R., Wang F., Muller S. (2019). Lysosomes as a therapeutic target. Nat. Rev. Drug Discov..

[157] Xue X., Liang X.J. (2012). Overcoming drug efflux-based multidrug resistance in cancer with nanotechnology. Chin. J. Cancer.

[158] Slastnikova T.A., Ulasov A.V., Rosenkranz A.A., Sobolev A.S. (2018). Targeted Intracellular Delivery of Antibodies: The State of the Art. Front. Pharm..

[159] Din F.U., Aman W., Ullah I., Qureshi O.S., Mustapha O., Shafique S., Zeb A. (2017). Effective use of nanocarriers as drug delivery systems for the treatment of selected tumors. Int. J. Nanomed..

[160] Wente S.R., Rout M.P. (2010). The nuclear pore complex and nuclear transport. Cold Spring Harb. Perspect. Biol..

[161] Tran E.J., King M.C., Corbett A.H. (2014). Macromolecular transport between the nucleus and the cytoplasm: Advances in mechanism and emerging links to disease. Biochim. Biophys. Acta (BBA) Mol. Cell Res..

[162] Das S.K., Menezes M.E., Bhatia S., Wang X.Y., Emdad L., Sarkar D., Fisher P.B. (2015). Gene Therapies for Cancer: Strategies, Challenges and Successes. J. Cell. Physiol..

[163] Cross D., Burmester J.K. (2006). Gene therapy for cancer treatment: Past, present and future. Clin. Med. Res..

[164] Hidai C., Kitano H. (2018). Nonviral gene therapy for cancer: A review. Diseases.

[165] Goswami R., Subramanian G., Silayeva L., Newkirk I., Doctor D., Chawla K., Chattopadhyay S., Chandra D., Chilukuri N., Betapudi V. (2019). Gene therapy leaves a vicious cycle. Front. Oncol..

[166] Shegokar R., Fernandes A.R., Souto E.B., Shegokar R., Souto E.B. (2018). Nanopharmaceuticals in immunology: What’s new in research?. Emerging Nanotechnologies in Immunology.

[167] Waldman A.D., Fritz J.M., Lenardo M.J. (2020). A guide to cancer immunotherapy: From T cell basic science to clinical practice. Nat. Rev. Immunol..

[168] Cruvinel W.D.M., Mesquita Júnior D., Araújo J.A.P., Catelan T.T.T., Souza A.W.S.D., Silva N.P.D., Andrade L.E.C. (2010). Immune system: Part I. Fundamentals of innate immunity with emphasis on molecular and cellular mechanisms of inflammatory response. Rev. Bras. Reumatol..

[169] Moore T.V., Lyons G.E., Brasic N., Roszkowski J.J., Voelkl S., Mackensen A., Kast W.M., Le Poole I.C., Nishimura M.I. (2009). Relationship between CD8-dependent antigen recognition, T cell functional avidity, and tumor cell recognition. Cancer Immunol. Immunother..

[170] Vera J.F., Brenner M.K., Dotti G. (2009). Immunotherapy of human cancers using gene modified T lymphocytes. Curr. Gene.

[171] Zappasodi R., Merghoub T., Wolchok J.D. (2018). Emerging Concepts for Immune Checkpoint Blockade-Based Combination Therapies. Cancer Cell.

[172] Barrueto L., Caminero F., Cash L., Makris C., Lamichhane P., Deshmukh R.R. (2020). Resistance to Checkpoint Inhibition in Cancer Immunotherapy. Transl. Oncol..

[173] Li X., Shao C., Shi Y., Han W. (2018). Lessons learned from the blockade of immune checkpoints in cancer immunotherapy. J. Hematol. Oncol..

[174] Cano A., Ettcheto M., Espina M., López-Machado A., Cajal Y., Rabanal F., Sánchez-López E., Camins A., García M.L., Souto E.B. (2020). State-of-the-art polymeric nanoparticles as promising therapeutic tools against human bacterial infections. J. Nanobiotechnol..

[175] Wagner A.M., Knipe J.M., Orive G., Peppas N.A. (2019). Quantum dots in biomedical applications. Acta Biomater..

[176] Kara H.E.Ş., Ertaş N. (2017). Quantum Dots for Pharmaceutical and Biomedical Analysis. Spectroscopic Analyses-Developments and Applications.

[177] Elhissi A.M.A., Ahmed W., Hassan I.U., Dhanak V.R., D’Emanuele A. (2012). Carbon nanotubes in cancer therapy and drug delivery. J. Drug Deliv..

[178] Jeevanandam J., Barhoum A. (2018). Review on nanoparticles and nanostructured materials: History, sources, toxicity and regulations. Beilstein J. Nanotechnol..

[179] Chen M., Wu F., Yu L., Cai Y., Chen H., Zhang M. (2019). Chloride binding capacity of LDHs with various divalent cations and divalent to trivalent cation ratios in different solutions. CrystEngComm.

[180] Zielińska A., Pereira I., Antunes S., Veiga F.J., Santos A.C., Nowak I., Silva A.M., Souto E.B., Grumezescu A.M. (2018). Mesoporous silica nanoparticles as drug delivery systems against melanoma. Design of Nanostructures for Theranostics Applications.

[181] Silva A.M., Alvarado H.L., Abrego G., Martins-Gomes C., Garduno-Ramirez M.L., Garcia M.L., Calpena A.C., Souto E.B. (2019). In Vitro Cytotoxicity of Oleanolic/Ursolic Acids-Loaded in PLGA Nanoparticles in Different Cell Lines. Pharmaceutics.

[182] Bozzuto G., Molinari A. (2015). Liposomes as nanomedical devices. Int. J. Nanomed..

[183] (2021). Let’s talk about lipid nanoparticles. Nat. Rev. Mater..

[184] Hong S., Choi D.W., Kim H.N., Park C.G., Lee W., Park H.H. (2020). Protein-Based Nanoparticles as Drug Delivery Systems. Pharmaceutics.

[185] Hanafy N.A.N., El-Kemary M., Leporatti S. (2018). Micelles Structure Development as a Strategy to Improve Smart Cancer Therapy. Cancers.

[186] Falzone L., Salomone S., Libra M. (2018). Evolution of Cancer Pharmacological Treatments at the Turn of the Third Millennium. Front. Pharm..

[187] Pucci C., Martinelli C., Ciofani G. (2019). Innovative approaches for cancer treatment: Current perspectives and new challenges. Ecancermedicalscience.

[188] Salama L., Pastor E.R., Stone T., Mousa S.A. (2020). Emerging Nanopharmaceuticals and Nanonutraceuticals in Cancer Management. Biomedicines.

[189] Souto E.B., Souto S.B., Campos J.R., Severino P., Pashirova T.N., Zakharova L.Y., Silva A.M., Durazzo A., Lucarini M., Izzo A.A. (2019). Nanoparticle Delivery Systems in the Treatment of Diabetes Complications. Molecules.

[190] Ren Z., Ma X., Duan Z., Chen X. (2020). Diagnosis, Therapy, and Prognosis for Hepatocellular Carcinoma. Anal. Cell. Pathol..

[191] Liao R., Zhang X.-D., Li G.-Z., Qin K.-L., Yan X. (2020). Comparison of transcatheter arterial chemoembolization with raltitrexed plus liposomal doxorubicin vs. tegafur plus pirarubicin for unresectable hepatocellular carcinoma. J. Gastrointest. Oncol..

[192] Wei M., Guo X., Tu L., Zou Q., Li Q., Tang C., Chen B., Xu Y., Wu C. (2015). Lactoferrin-modified PEGylated liposomes loaded with doxorubicin for targeting delivery to hepatocellular carcinoma. Int. J. Nanomed..

[193] Li X., Diao W., Xue H., Wu F., Wang W., Jiang B., Bai J., Lian B., Feng W., Sun T. (2020). Improved efficacy of doxorubicin delivery by a novel dual-ligand-modified liposome in hepatocellular carcinoma. Cancer Lett..

[194] Gong C., Hu K., Wang X., Wangyang P., Yan C., Chu J., Liao M., Dai L., Zhai T., Wang C. (2018). 2D Nanomaterial Arrays for Electronics and Optoelectronics. Adv. Funct. Mater..

[195] Yahya E.B., Alqadhi A.M. (2021). Recent trends in cancer therapy: A review on the current state of gene delivery. Life Sci..

[196] Severino P., Szymanski M., Favaro M., Azzoni A.R., Chaud M.V., Santana M.H., Silva A.M., Souto E.B. (2015). Development and characterization of a cationic lipid nanocarrier as non-viral vector for gene therapy. Eur J Pharm Sci.

[197] Amreddy N., Babu A., Muralidharan R., Panneerselvam J., Srivastava A., Ahmed R., Mehta M., Munshi A., Ramesh R. (2018). Recent advances in nanoparticle-based cancer drug and gene delivery. Adv. Cancer Res..

[198] Thakkar S., Sharma D., Kalia K., Tekade R.K. (2020). Tumor microenvironment targeted nanotherapeutics for cancer therapy and diagnosis: A review. Acta Biomater..

[199] Shi J., Kantoff P.W., Wooster R., Farokhzad O.C. (2017). Cancer nanomedicine: Progress, challenges and opportunities. Nat. Rev. Cancer.

[200] Ediriwickrema A., Saltzman W.M. (2015). Nanotherapy for cancer: Targeting and multifunctionality in the future of cancer therapies. ACS Biomater. Sci. Eng..

[201] Song W., Das M., Chen X. (2020). Nanotherapeutics for immuno-oncology: A crossroad for new paradigms. Trends Cancer.

[202] Karimi H., Soleimanjahi H., Abdoli A., Banijamali R.S. (2020). Combination therapy using human papillomavirus L1/E6/E7 genes and archaeosome: A nanovaccine confer immuneadjuvanting effects to fight cervical cancer. Sci. Rep..

[203] Kokkinos J., Ignacio R.M.C., Sharbeen G., Boyer C., Gonzales-Aloy E., Goldstein D., McCarroll J.A., Phillips P.A., Initiative A.P.C.G. (2020). Targeting the Undruggable in pancreatic Cancer using Nano-based gene silencing drugs. Biomaterials.

[204] Mainini F., Eccles M.R. (2020). Lipid and Polymer-Based Nanoparticle siRNA Delivery Systems for Cancer Therapy. Molecules.

[205] Ingram N., McVeigh L.E., Abou-Saleh R.H., Maynard J., Peyman S.A., McLaughlan J.R., Fairclough M., Marston G., Valleley E.M., Jimenez-Macias J.L. (2020). Ultrasound-triggered therapeutic microbubbles enhance the efficacy of cytotoxic drugs by increasing circulation and tumor drug accumulation and limiting bioavailability and toxicity in normal tissues. Theranostics.

[206] Tian B., Hua S., Liu J. (2020). Cyclodextrin-based delivery systems for chemotherapeutic anticancer drugs: A review. Carbohydr. Polym..

[207] Yassemi A., Kashanian S., Zhaleh H. (2020). Folic acid receptor-targeted solid lipid nanoparticles to enhance cytotoxicity of letrozole through induction of caspase-3 dependent-apoptosis for breast cancer treatment. Pharm. Dev. Technol..

[208] Zhong H.H., Wang H.Y., Li J., Huang Y.Z. (2019). TRAIL-based gene delivery and therapeutic strategies. Acta Pharmacol. Sin..

[209] Chen X., Xie D., Zhao Q., You Z.H. (2019). MicroRNAs and complex diseases: From experimental results to computational models. Brief. Bioinform..

[210] Chen X., Tang W.J., Shi J.B., Liu M.M., Liu X.H. (2020). Therapeutic strategies for targeting telomerase in cancer. Med. Res. Rev..

[211] Guterres A.N., Villanueva J. (2020). Targeting telomerase for cancer therapy. Oncogene.

[212] Maurer S., Salih H.R., Smirnow I., Lauer U.M., Berchtold S. (2019). Suicide gene-armed measles vaccine virus for the treatment of AML. Int. J. Oncol..

[213] Hossain J.A., Riecken K., Miletic H., Fehse B. (2019). Cancer Suicide Gene Therapy with TK.007. Methods Mol. Biol..

[214] Cáceres B., Ramirez A., Carrillo E., Jimenez G., Griñán-Lisón C., López-Ruiz E., Jiménez-Martínez Y., Marchal J.A., Boulaiz H. (2019). Deciphering the Mechanism of Action Involved in Enhanced Suicide Gene Colon Cancer Cell Killer Effect Mediated by Gef and Apoptin. Cancers.

[215] Beg S., Alharbi K.S., Alruwaili N.K., Alotaibi N.H., Almalki W.H., Alenezi S.K., Altowayan W.M., Alshammari M.S., Rahman M. (2020). Nanotherapeutic systems for delivering cancer vaccines: Recent advances. Nanomedicine.

[216] Lu Y., Wu F., Duan W., Mu X., Fang S., Lu N., Zhou X., Kong W. (2020). Engineering a “PEG-g-PEI/DNA nanoparticle-in-PLGA microsphere” hybrid controlled release system to enhance immunogenicity of DNA vaccine. Mater. Sci. Eng. C Mater. Biol. Appl..

[217] Gonçalves M., Mignani S., Rodrigues J., Tomás H. (2020). A glance over doxorubicin based-nanotherapeutics: From proof-of-concept studies to solutions in the market. J. Control. Release Off. J. Control. Release Soc..

[218] Ali E.S., Sharker S.M., Islam M.T., Khan I.N., Shaw S., Rahman M.A., Uddin S.J., Shill M.C., Rehman S., Das N. (2021). Targeting cancer cells with nanotherapeutics and nanodiagnostics: Current status and future perspectives. Semin. Cancer Biol..

[219] Xu F., Xia Q., Wang P. (2020). Rationally Designed DNA Nanostructures for Drug Delivery. Front. Chem..

[220] Cao Y., Huang H.Y., Chen L.Q., Du H.H., Cui J.H., Zhang L.W., Lee B.J., Cao Q.R. (2019). Enhanced Lysosomal Escape of pH-Responsive Polyethylenimine-Betaine Functionalized Carbon Nanotube for the Codelivery of Survivin Small Interfering RNA and Doxorubicin. ACS Appl Mater Interfaces.

[221] Zhao Y., Zhao X., Cheng Y., Guo X., Yuan W. (2018). Iron Oxide Nanoparticles-Based Vaccine Delivery for Cancer Treatment. Mol. Pharm..

[222] Bartelds R., Nematollahi M.H., Pols T., Stuart M.C.A., Pardakhty A., Asadikaram G., Poolman B. (2018). Niosomes, an alternative for liposomal delivery. PLoS ONE.

[223] Waglewska E., Pucek-Kaczmarek A., Bazylińska U. (2020). Novel Surface-Modified Bilosomes as Functional and Biocompatible Nanocarriers of Hybrid Compounds. Nanomaterials.

[224] Ahmed S., Kassem M.A., Sayed S. (2020). Bilosomes as Promising Nanovesicular Carriers for Improved Transdermal Delivery: Construction, in vitro Optimization, ex vivo Permeation and in vivo Evaluation. Int. J. Nanomed..

[225] Kumar G., Nandakumar K., Mutalik S., Rao C.M. (2020). Biologicals to direct nanotherapeutics towards HER2-positive breast cancers. Nanomedicine.

[226] Lv C., Zhang T.Y., Lin Y., Tang M., Zhai C.H., Xia H.F., Wang J., Zhang Z.L., Xie Z.X., Chen G. (2019). Transformation of Viral Light Particles into Near-Infrared Fluorescence Quantum Dot-Labeled Active Tumor-Targeting Nanovectors for Drug Delivery. Nano Lett..

[227] Zhang Q., Wu W., Zhang J., Xia X. (2020). Merits of the ‘good’ viruses: The potential of virus-based therapeutics. Expert Opin. Biol. Ther..

[228] Yang F., Zhao Z., Sun B., Chen Q., Sun J., He Z., Luo C. (2020). Nanotherapeutics for Antimetastatic Treatment. Trends Cancer.

[229] Young S.W., Stenzel M., Yang J.L. (2016). Nanoparticle-siRNA: A potential cancer therapy?. Crit. Rev. Oncol. Hematol..

[230] Liu T.I., Lu T.Y., Yang Y.C., Chang S.H., Chen H.H., Lu I.L., Sabu A., Chiu H.C. (2020). New combination treatment from ROS-Induced sensitized radiotherapy with nanophototherapeutics to fully eradicate orthotopic breast cancer and inhibit metastasis. Biomaterials.

[231] Heuts J., Varypataki E.M., van der Maaden K., Romeijn S., Drijfhout J.W., van Scheltinga A.T., Ossendorp F., Jiskoot W. (2018). Cationic Liposomes: A Flexible Vaccine Delivery System for Physicochemically Diverse Antigenic Peptides. Pharm. Res..

[232] Biswas A., Bayer I.S., Biris A.S., Wang T., Dervishi E., Faupel F. (2012). Advances in top–down and bottom–up surface nanofabrication: Techniques, applications & future prospects. Adv. Colloid Interface Sci..

[233] Souto E.B., Silva G.F., Dias-Ferreira J., Zielinska A., Ventura F., Durazzo A., Lucarini M., Novellino E., Santini A. (2020). Nanopharmaceutics: Part II—Production scales and clinically compliant production methods. Nanomaterials.

[234] Tutar R., Motealleh A., Khademhosseini A., Kehr N.S. (2019). Functional Nanomaterials on 2D Surfaces and in 3D Nanocomposite Hydrogels for Biomedical Applications. Adv. Funct. Mater..

[235] Lee W., Liu Y., Lee Y., Sharma B.K., Shinde S.M., Kim S.D., Nan K., Yan Z., Han M., Huang Y. (2018). Two-dimensional materials in functional three-dimensional architectures with applications in photodetection and imaging. Nat. Commun..

[236] Soares S., Sousa J., Pais A., Vitorino C. (2018). Nanomedicine: Principles, Properties, and Regulatory Issues. Front. Chem..

[237] Hua S., de Matos M.B.C., Metselaar J.M., Storm G. (2018). Current Trends and Challenges in the Clinical Translation of Nanoparticulate Nanomedicines: Pathways for Translational Development and Commercialization. Front. Pharm..

[238] Halamoda-Kenzaoui B., Box H., van Elk M., Gaitan S., Geertsma R., Gainza Lafuente E., Owen A., Del Pozo A., Roesslein M., Bremer-Hoffmann S. (2019). Anticipation of Regulatory Needs for Nanotechnology-Enabled Health Products.

